# Arrested in Glass: Actin within Sophisticated Architectures of Biosilica in Sponges

**DOI:** 10.1002/advs.202105059

**Published:** 2022-02-13

**Authors:** Hermann Ehrlich, Magdalena Luczak, Rustam Ziganshin, Ivan Mikšík, Marcin Wysokowski, Paul Simon, Irena Baranowska‐Bosiacka, Patrycja Kupnicka, Alexander Ereskovsky, Roberta Galli, Sergey Dyshlovoy, Jonas Fischer, Konstantin R. Tabachnick, Iaroslav Petrenko, Teofil Jesionowski, Anna Lubkowska, Marek Figlerowicz, Viatcheslav N. Ivanenko, Adam P. Summers

**Affiliations:** ^1^ Institute of Electronic and Sensor Materials TU Bergakademie Freiberg Freiberg 09599 Germany; ^2^ Center for Advanced Technology Adam Mickiewicz University Poznan 61614 Poland; ^3^ Institute of Bioorganic Chemistry Polish Academy of Sciences Poznan 61704 Poland; ^4^ Institute of Bioorganic Chemistry Russian Academy of Sciences Moscow 142290 Russian Federation; ^5^ Institute of Physiology The Czech Academy of Sciences Prague 142 20 Czech Republic; ^6^ Faculty of Chemical Technology Institute of Chemical Technology and Engineering Poznan University of Technology Poznan 60965 Poland; ^7^ Max Planck Institute for Chemical Physics of Solids Dresden 01187 Germany; ^8^ Department of Biochemistry and Medical Chemistry Pomeranian Medical University in Szczecin Szczecin 70111 Poland; ^9^ Institut Méditerranéen de Biodiversité et d'Ecologie (IMBE) CNRS IRD Aix Marseille Université Marseille 13003 France; ^10^ Biological Faculty St. Petersburg State University St. Petersburg 199034 Russian Federation; ^11^ Koltzov Institute of Developmental Biology of Russian Academy of Sciences Moscow 119334 Russian Federation; ^12^ Clinical Sensoring and Monitoring Department of Anesthesiology and Intensive Care Medicine TU Dresden Dresden 01307 Germany; ^13^ Laboratory of Experimental Oncology University Medical Center Hamburg‐Eppendorf Hamburg 20251 Germany; ^14^ Laboratory of Pharmacology A.V. Zhirmunsky National Scientific Center of Marine Biology Far Eastern Branch Russian Academy of Sciences Vladivostok 690041 Russian Federation; ^15^ Shirshov Institute of Oceanology of Russian Academy of Sciences Moscow 117218 Russian Federation; ^16^ Department of Functional Diagnostics and Physical Medicine Faculty of Health Sciences Pomeranian Medical University in Szczecin Szczecin 71210 Poland; ^17^ Department of Invertebrate Zoology Biological Faculty Lomonosov Moscow State University Moscow 119991 Russian Federation; ^18^ Department of Biology Friday Harbor Labs University of Washington Friday Harbor WA 98195 USA

**Keywords:** actin, biological materials, biomineralization, biosilica, sponges

## Abstract

Actin is a fundamental member of an ancient superfamily of structural intracellular proteins and plays a crucial role in cytoskeleton dynamics, ciliogenesis, phagocytosis, and force generation in both prokaryotes and eukaryotes. It is shown that actin has another function in metazoans: patterning biosilica deposition, a role that has spanned over 500 million years. Species of glass sponges (Hexactinellida) and demosponges (Demospongiae), representatives of the first metazoans, with a broad diversity of skeletal structures with hierarchical architecture unchanged since the late Precambrian, are studied. By etching their skeletons, organic templates dominated by individual F‐actin filaments, including branched fibers and the longest, thickest actin fiber bundles ever reported, are isolated. It is proposed that these actin‐rich filaments are not the primary site of biosilicification, but this highly sophisticated and multi‐scale form of biomineralization in metazoans is ptterned.

## Graphical abstract

This work provides an answer to one of the fundamental questions of science, namely, why biological glass grows past at ambient temperatures and reaches dimensions in the meter range. It is shown that actin has a new function in metazoans: patterning highly sophisticated biosilica deposition in sponges that has spanned over 500 million years.

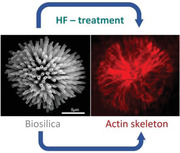

## 1 Introduction

Sponges (Porifera) are the sister‐group to all other animals,^[^ ^1^, ^2^, ^3^ ^]^ with fossils dating to the Late Protoerozoic.^[^ ^4^ ^]^ These exclusively aquatic, filter‐feeding organisms have persisted with little morphological change for hundreds of millions of years, likely due to their ability to produce biomineralized, mechanically robust three dimensional (3D) skeletal constructs, as well as synthesizing a broad variety of secondary metabolites with antibiotic and cytotoxic properties.^[^ ^5^, ^6^ ^]^ The inorganic chemistry of poriferan skeletons with respect to silica in Hexactinellida, Demospongiae, and Homoscleromorpha, or calcium carbonates in Calcarea, is well understood, but the role of the bio‐organic phases in skeletogenesis of sponges is still debated.^[^ ^7^, ^8^, ^9^, ^10^ ^]^ Silica‐based structures in sponges have several roles, including protection, support of the body form, maintenance of posture in flow, as well as anchoring to sandy and muddy bottoms. The main players in poriferan biosilicification and spiculogenesis are low molecular weight proteins (i.e., silicateins, cathepsins)^[^ ^10^, ^11^, ^12^ ^]^ in demosponges, accompanied by glassin,^[^ ^13^ ^]^ collagen,^[^ ^14^ ^]^ and chitin^[^ ^15^, ^16^ ^]^ in glass sponges (Hexactinellida). Though their presence during skeletal formation has been confirmed, there remain questions of pattern drivers that lead to the diversity of shape (more than 80 and 46 morphotypes in Demospongiae and Hexactinellida, respectively),^[^ ^5^, ^17^ ^]^ size (from micrometer‐ to up to 3 meter‐long),^[^ ^6^ ^]^ network connectivity (monaxons, triaxons, tetraxons),^[^ ^18^, ^19^ ^]^ and superficial ornamentation. Intriguingly, all these biosilica‐based structures possess a common feature: a proteinaceous axial filament situated inside the axial channels distributed along the rays of spicules. This organic matter has nanocrystalline‐like properties^[^ ^7^, ^19^ ^]^ and is found in axial channels of both structurally simple and hierarchically complex poriferan biosilica (see Figures S2–S4, S10, and S11, Supporting Information) in the form of linear, bifurcated, or branched microfilaments. They are not “cemented inside,” but lie freely in the channels (Figures S1 and S2, Supporting Information). This is in contrast to the mineralization‐associated molecules such as silicateins, which are not branched, and are tightly bound in the biomineralized structure where they catalyze and template silica.^[^ ^10^ ^]^

The diversity of forms of biosilica is exemplified by the extreme case of *Monorhaphis* sponges, which can produce meter‐length spicules (Figures S26 and S27, Supporting Information). Our work provides an answer to one of the fundamental questions of biomaterials science, namely, how this “biological glass” grows at ambient temperatures (from ‐1.9°C to 24°C) to reach such great lengths. We will show that axial filaments of both hexactinellids and demosponges, first revealed by electron microscopy,^[^ ^20^, ^21^ ^]^ are principal instances of actin in a newly discovered role: patterning silica architecture of metazoans in vivo.

## 2 Results and discussion

### 2.1 Actin in the Skeletal Structures of Sponges

Previously, we identified highly hydroxylated collagen as a template for biosilicification in the meter‐long anchoring spicules of the glass sponge *Hyalonema sieboldii*,^[^ ^14^ ^]^ there was also actin in these extracts which we attributed at that time to cytoskeletal remnants. In hexactinellid sponges, there are 0.5 mm long actin microfilaments in adherent tissues and filopodia.^[^ ^22^, ^23^ ^]^ Dense tracts of actin have been also confirmed in endopinacocytes of the apical pinacoderm, canals, and the osculum of the freshwater demosponge *Ephydatia muelleri*.^[^ ^24^, ^25^ ^]^ Since actin has traditionally been perceived as a strictly intracellular protein, it has not been predicted in such structures as spicules, or skeletal frameworks.

We developed a new method that works at a microscopic scale and isolates axial filaments from siliceous microstructures derived from diverse species of sponges. The approach is based on the gentle application of a drop of 10% hydrofluoric acid (HF) onto the surface of a spicule placed on an inclined plastic surface. This acid dissolves biosilica leaving the organic matrix intact (Figure S1a, Supporting Information).

The use of HF is a traditional and proven method in the study of sponges for dissolving spicules, consisting mainly of glassy amorphous hydrated silica similar to opal or silica gel. This method has proven itself in cytology, developmental biology, molecular biology, as well as biochemistry of sponges.^[^ ^21^, ^26^, ^27^, ^28^, ^29^ ^]^ This dilute HF does not have negative effects on cells, extracellular matrix, or the internal organic structures of spicules. Furthermore, our SEM and TEM observations revealed no effect of HF on the organization or integrity of actin in our samples. Similar results have been obtained on diverse biological systems by other authors.^[^ ^30^, ^31^, ^32^ ^]^

Following this HF‐ based treatment, residual axial filaments remain strongly attached to the plastic surface and can be rinsed with water and identified using special phalloidin reagents, or immunostaining. Our method shows axial filaments fixed on the plastic surface can also be found in partially demineralized structures (Figure S1 and the legend to this, Supporting Information), eliminates the possibility of external contamination of the sample with actin fibers of cellular origin. Drying of the specimen during the phalloidin staining fixes the actin filament, formerly sheathed in biosilica to the slide for further investigation.

**Figure 1:**
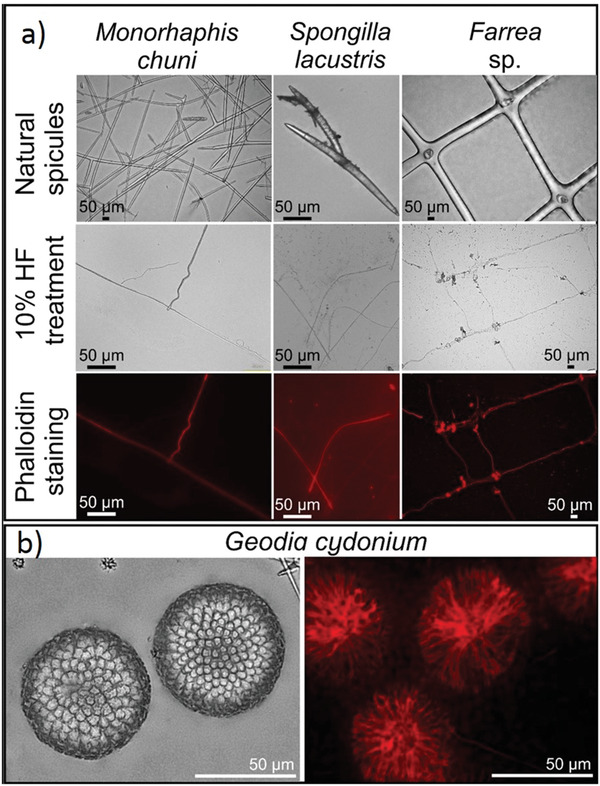
Identification of actin in the axial filaments of diverse sponge species using iFluor 594‐Phalloidin red stain. a) Overview of stained axial filaments isolated from selected biosilica‐based structures such as tauactines of *M. chuni* (Hexactinellida), megascleres (oxea) of *S. lacustris* (Demospongiae), and skeletal framework of *Farrea*. sp. (Hexactinellida) following 10% HF treatment. All the axial filaments, isolated from both marine (*M. chuni, Farrea* sp.) and freshwater (*S. lacustris*) sponges, resemble the size and morphology of the siliceous skeletal structures they were derived from. b) HF‐based treatment of siliceous spherical microspined rays forming the oxyasters of *G. cydonium* marine demosponge led to the isolation of an organic matrix with branching, radially spaced microfibers also visible after iFluor 594‐Phalloidin staining (see also Figure S10, Supporting Information).

**Figure 2:**
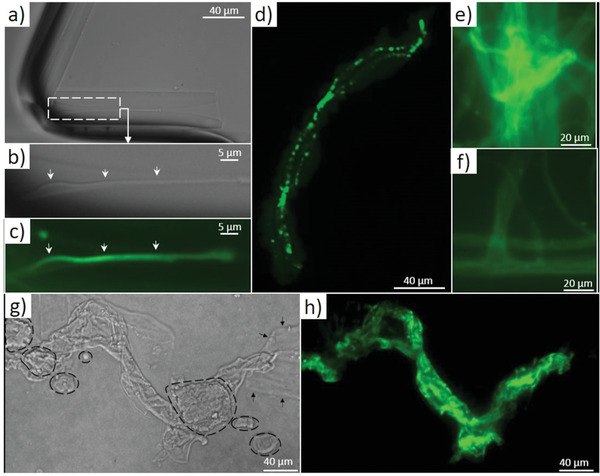
Confirmation of the presence of actin in axial filaments of selected sponges species using immunofluorescent analysis with primary anti‐*β*‐actin antibody and secondary (anti‐rabbit IgG (H+L), F(ab′)2 Fragment (Alexa Fluor 488 Conjugate) antibodies. a) Light microscopy image of the partially demineralized spicule of the marine demosponge *G. cydonium* after immunostaining. The location of the axial filament in the axial channel of the spicule is visible with both b, arrows) light microscopy and c, arrows) fluorescence. d) The bundle of self‐aggregated axial filaments isolated from spicules of *S. lacustris* purified with HNO_3_ after immunostaining. e) Fluorescence microscopy of axial filaments isolated from spicules (known as tauactines) of *M. chuni* (see also Figure 1a). f) Treatment of axial filaments only with secondary antibodies shows weak autofluorescence in comparison to (e). g,h) Immunostaining confirms the location of actin in the partially demineralized *Hyalonema populiferum*. g) Fragments of residual silica (dotted lines) are detectable as well as demineralized residual organic matrix (arrows) that did not immunostain, in contrast to h) the axial filament.

With this approach we identified actin in the axial filaments of representatives of Hexactinellida (*Aphrocallistes beatrix*, *Asconema setubalense*, *Caulophacus arcticus*, *Hyalonema* (*Corynonema*) *populiferum*, *Farrea* sp., *Euplectella aspergillum*, *Malacosaccus* sp., *Monorhaphis chuni*, *Rossella* *antarctica*, *Walteria flemmingi*) (**Figures** **1** and **2**a and Figures S5–S8, Supporting Information) and Demospongiae (*Cladorhiza corona*, *Cinachyra antarctica*, *Spongilla lacustris*, *Geodia cydonium*) (Figure 1, and Figures S5, S9, and S10, Supporting Information) using conventional phalloidin staining^.[^ ^33^, ^34^ ^]^ Actin was found in the axial filaments of this diversity of spicules, including those with unusual and very complex spiny microarchitecture such as the demosponge *G. cydonium* (Figure 1b, and Figure S10, Supporting Information), and sponges with hierarchical, geometric, skeletal designs such as the glass sponges *Farrea* sp. (Figure 1a), *A. beatrix* (Figure S8, Supporting Information), and *E. aspergillum* (Figure S11, Supporting Information).

To confirm the presence of actin in axial filaments of selected sponges, we used immunostaining with primary and secondary ß‐actin antibodies. Fluorescently labelled secondary antibodies were found in partially demineralized spicules of the demosponge *G. cydonium* (Figure 2c) and the glass sponge *Hyalonema populiferum* (Figure 2h) as well as in isolated, fully demineralized, axial filaments of the demosponge *S. lacustis* (Figure 2d), and the glass sponge *M. chuni* (Figure 2e).

The dimensions of the observed fibers stained with ß‐actin antibodies with respect to both diameter and length are unambiguously larger than any intracellular actins ever described, probably, due to possible aggregation during processing (see also Figures S12–S14, Supporting Information).

### 2.2 Proteomics Reveals Actin as a Component of Axial Filaments

**Figure 3:**
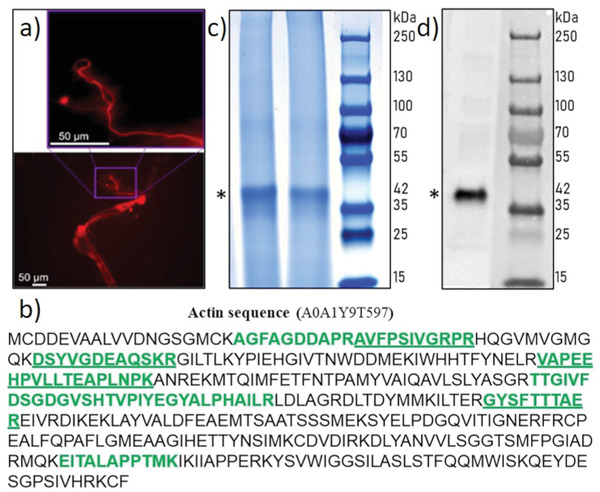
Identification of actin in the axial filament of the glass sponge *Asconema setubalense*. a) iFluor 594‐Phalloidin red staining of partially demineralized spicules shows the axial filament. b) The amino acid sequence of actin (UniProt ID A0A1Y9T597) was identified by the MS proteomic approach in the axial filament of *A. setubalense*. Seven peptides were identified using an in‐solution digestion and label‐free nanoLC‐MS/MS approach (marked in bold), yielding 27.1% coverage of a protein sequence (Table S1, Supporting Information). c) SDS‐PAGE analysis of the *A. setubalense* actin filaments indicating the presence of 42 kDa band visualized by Coomassie blue G‐250 staining (1# and 2# marked by an asterisk). Lanes #1 and #2 correspond to axial filaments isolated from two different specimens of the same sponge. Four actin peptides were found in this band by MS protein identification (bold and underlined peptides), with 13% sequence coverage (Table S1, Supporting Information). d) Western blot analysis with a human anti‐ß‐actin antibody confirmed the actin signal at 42 kDa (marked by an asterisk).

The expression of actin genes and the formation of actin filaments in specialized cells and tissues of sponges are well documented.^[^ ^22^, ^23^, ^35^ ^]^ However, there is presently no evidence to indicate whether these cells and consequently actin filaments, are present in sponge spicules, which are formed in cells called sclerocytes (see note 13, Supporting Information). To address this question, we analysed axial filaments extracted from spicules of diverse sponge species with complementary proteomic approaches using SDS‐PAGE and LC separation, followed by mass spectrometry and western blot analysis (for details see notes 5 and 6, Supporting Information). These methods showed actin in the axial filament from spicules of the glass sponges *A. setubalense* (**Figure** **3**b–d, Table S1, Supporting Information), *M. chuni* (Table S2, Figure S16, Supporting Information), *Monorhaphis* sp. (Table S3, Figure S16, Supporting Information), and *C. arcticus* (Figures S15 and S16; Table S4, Supporting Information). In particular, 7, 14, 15, and 21 actin peptides were identified utilizing LC‐MS/MS for *A. setubalense*, *M. chuni*, *M. sp* and *C. arcticus* samples, respectively, yielding 13–56.4% coverage of actin sequence. For *A. setubalense* sponge presence of actin was additionally corroborated by western blot using a specific anti‐ß‐actin antibody (Figure 3d).

We used the same spectrum of proteomic methods to analyze axial filaments isolated from spicules of the freshwater demosponge *S. lacustris* and found actin in association with silicateins (Figures S15–S17; Table S5, Supporting Information). Three actin and four silicatein peptides were identified in these samples with 12% and 19% sequence coverage, respectively. Actin in *S. lacustris* was also validated with Western blot using an anti‐ß‐actin antibody. These results demonstrate the role of actin as a core around which silicateins self‐assemble and act as biomineralizing agents.^[^ ^11^, ^12^ ^]^

### 2.3 Identification of Actin‐Like Structures in Axial Filaments

**Figure 4:**
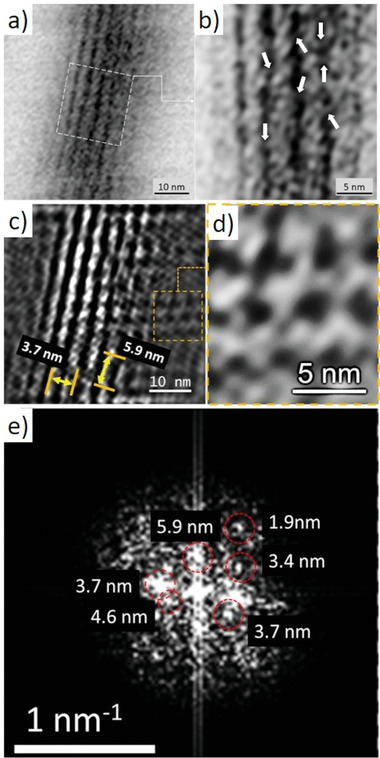
TEM imagery and Fourier analysis of the axial filaments isolated from spicules of the glass sponge *M. chuni*. b) Zoomed image of a) the TEM image that represents nanofibrillar organization of the selected area of isolated axial filament of *M. chuni* shows the cross‐linked nanoarchitecture (arrows) typical for F‐actin filaments, which form right‐handed, parallel, and staggered structures in all eukaryotes.^[^ ^38^, ^41^ ^]^ The FFT in (e) taken from (d) indicates different large periodicities typical for actin such as 5.9, 4.6, 3.7, 3.4, and 1.9 nm (see for details including statistical analysis Table S7, Supporting Information). Individual reflections are shown with red colored dotted lines. d) The axial filament lattice shows high similarity to that of actin standards reported by other authors.^[^ ^37^, ^40^ ^]^

We used high‐resolution transmission electron microscopy (HR‐TEM) to visualize the characteristic periodic structure of actin,^[^ ^36^, ^37^, ^38^ ^]^ and to resolve actin structure and assemblies. A fast Fourier transform (FFT) image processing technique was informative for determining the crystal structure of HRTEM images in reciprocal space. We prepared specimens for ultrathin‐sections of transmission electron microscopy (Figures S18–S21, Supporting Information), and imaged them for characteristic Fourier patterns (**Figure** **4**e) from processed high‐resolution brightfield images, as well as to visualize actin filaments (Figure 4b–d). The Fourier pattern of the axial filaments, isolated from hexactinellids (i.e., *M. chuni* Figure 4e; Figure S21a, and *C. arcticus* Figure S22d,h, Supporting Information), matched with that of F‐actin (see lattice parameters in Table S7, Supporting Information). Also, the nanostructural organization of axial filaments (Figure 4c and Figures S18–S20, Supporting Information) is typical of F‐actin from other sources.^[^ ^39^, ^40^, ^41^ ^]^ The logical question of whether they are branched filament networks (Arp2/3 mediated) or bundled filaments (formin‐mediated) should be answered in the future experiments.

We used Raman spectroscopy, a high precision method of spectroscopic characterization, on axial filaments isolated from sponges including *M. chuni* and *C. arcticus*. Raman spectra of reference actin from rabbit muscle matched those from the axial filaments, and they differ from diverse non‐actin‐related proteins (i.e., silicatein A1, collagen, pepsin, trypsin, etc.) (Figure S23 and Table S8, Supporting Information).

### 2.4 Actin Inhibition Prevents Spicule Formation

Tesson & Hildebrand^[^ ^42^ ^]^ showed that actin filaments play a substantial role in the formation of meso‐ and micro‐scale silica structure in the frustule formation of diatoms. One role is in defining microscale processes such as the size and shape of the silica deposition vesicles and the leading edge of the silicification front. Actin was also visualized in diatoms *Cyclotella cryptica*: an actin ring defined the full extent of the valves, and another concentration of actin filaments was associated with the growing front of silica deposition.^[^ ^43^ ^]^ There are also a number of inhibitor studies, including Latrunculin and direct microscopic observations that have shown actin plays an important role in shaping microscale silica structure in unicellular organisms (i.e., diatoms). (See for details^[^ ^44^, ^45^, ^46^, ^47^, ^48^ ^]^).

Latrunculin B, originally discovered in sponges, inhibits actin polymerization and is highly specific, and does not kill or stop cell function.^[^ ^49^, ^50^ ^]^ The evidence that the inhibition of F‐actin by Latrunculin is reversible is from cultured vertebrate cells^[^ ^51^ ^]^ and corals.^[^ ^52^ ^]^ Depolymerization or inhibition of actin filaments can prevent or slow down the secretion of certain protein vesicles and biomineralized elements. However, these elements can be produced and accumulated inside of the cells, as was shown for fully glycosylated invertase in yeasts,^[^ ^53^ ^]^ and silica scales in the haptophyte *Prymnesium neolepis*.^[^ ^49^ ^]^

**Figure 5:**
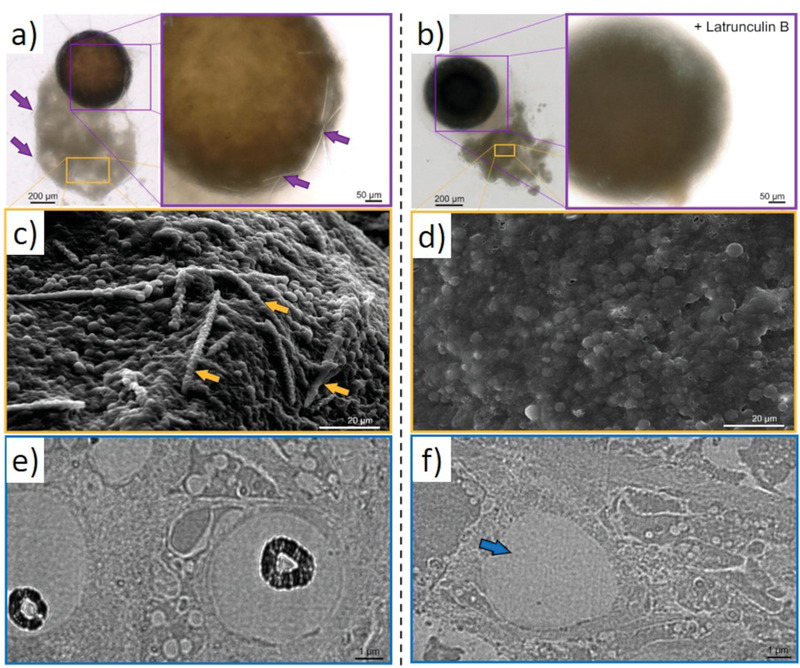
Latrunculin B‐mediated inhibition of siliceous spicules formation in *S. lacustris* demosponge. a) Light microscopy and SEM images of young sponge observed after hatching of gemmules under natural conditions shows the presence of c, arrows) glassy spicules. b) However, no spicules were observed in young sponges after the cultivation of the gemmules in the presence of latrunculin B (SEM image D), a well‐known inhibitor of actin polymerization in vivo (see also Figure S24, Supporting Information). TEM images of corresponding thin sections of sclerocytes confirm these observations and show f, arrow) the absence of spicules after treatment of gemmules with Latrunculin B in contrast to e) typical spicules formation under natural conditions (black, electron‐dense structures of biosilica, which surround the axial filaments of young spicules).

Consequently, we explored the role of actin in the development of spicules with an in vivo experiment on *S. lacustris* demosponge in which we inhibited actin formation. We treated the hatching gemmules with Latrunculin B, which binds to actin monomers and inhibits F‐actin polymerization^[^ ^51^, ^54^ ^]^ leading to F‐actin depolymerization.^[^ ^51^ ^]^ In samples treated with Latrunculin B, the young sponges grew, but siliceous spicules never appeared (**Figure** **5**b,d,f, and Figure S24, Supporting Information). Controls treated with tap water and DMSO as controls for solvent effects, divided normally, with no apparent effect on spiculogenesis (Figure 5a and Figure S24, Supporting Information). We infer that inhibition of actin polymerization interferes with spiculogenesis at a very early stage of mineralization. Our results echo those of Durak et al.^[^ ^49^ ^]^ who showed that disruption of the actin network interferes with the secretion of biomineralized elements in the marine unicellular silicifying haptophyte *P. neolepis*. Deliberately, we also used a concentration of Latrunculin B (1µM) similar to that used in the work with this unicellular biomineralizer.^[^ ^49^ ^]^ Additionally, it has been reported that F‐actin structures are involved in shaping and controlling mineralization by acting as a support for the dynamic templates involved in foraminifera biomineralization.^[^ ^55^ ^]^ We propose that actin filaments are the patterning template that controls the structure, shape, and size of siliceous spicules and distributes biomolecules (i.e., silicateins), responsible for the silicic acid polymerization in sponges. Detailed, comparative studies on the influence of different concentrations of Latrunculin B as well as other potent inhibitors (i.e., cytochalasins) of actin polymerization in skeletal structures of sponges should be carried out.

The duration of the experiments predicated on the development of time of *S. lacustris* sponges after their release from the gemmules, and the time required for the development of spicules under normal conditions. Recovery experiments were not necessary as cells were observed daily to be both live and unlysed via light microscopy (Figure 5).

We confirmed the role of actin in pattern formation by manipulating the ratio of Germanium (Ge) to Silicon (Si) during spicule morphogenesis.^[^ ^54^ ^]^ We added Ge to the growth medium of *S. lacustris* gemmules leading to young sponges with malformed spicules showing characteristic bulbs (Figure S25, Supporting Information). Fluorescence microscopy of these bulbs confirms that formation is associated with bifurcation of the actin‐based axial filament (Figure 1). In contrast to such fibrillar proteins as collagen, structural bifurcation and branching are recognized as one of the characteristic features of the actin filament (see for review^[^ ^55^ ^]^).

## 3 Conclusion

We confirmed the presence of actin in the silicified skeletal constructs of selected sponges species from both cold water (‐1.9°C – 4°C) (*A. beatrix*; *A. setubalense, C. arcticus*; *E. subarea*; *Farrea* sp., *H. (Corynonema) populiferum*, *Hyalonema* sp. *Malacosaccus* sp.;*M.chuni*; *R. antarctica*, *W. flemmingi*, *C, corona*) and warm water (20°C–24°C) (*S. lacustris*. *P. ficiformis*, *G. cydonium*) environments. The wide temperature range is further confirmation that actin, in the form of actin‐rich axial filaments, in Hexactinellid and Demospongid sponges, with both loose and fused skeletons, is fundamental to pattern generation. This is especially exciting because of the amazing structural diversity of their biosilica‐based skeletons.^[^ ^7^, ^17^, ^18^ ^]^

The occurrence of actin in skeletal constructs of other poriferan taxa, such as Homoscleromorpha and Calcarea, should be surveyed to confirm the generality of this result. In microscleres, there are actin‐rich axial filaments at the nanoscale in structures with simple and very complex (dichotomic, or polybranched) microarchitecture (Figure 1b and Figure S10, Supporting Information). But, in species of hexactinellids with large size body, and correspondingly large skeletons (i.e., Lanuginelline sponges that reach over 3.5 m in length, 2.0 m in height, and 1.5 m in width, or *Monorhaphis* with 3‐meter long individual spicules^[^ ^7^ ^]^) the axial filament is made up of actin bundles of remarkable size (Figures S18–S21, Supporting Information). These 3 mm thick, and up to 3‐meter long, axial filaments constructs are the largest actin filament‐based bundles ever reported (Figures S26 and S27, Supporting Information).

Since axial filaments inside the spicules are found unbound to the mineralized phase^[^ ^18^ ^]^ (Figure S2, Supporting Information), we assume that F‐actin here plays no direct role in biosilicification.

This raises the question ’*what is the mechanism behind actin driven pattern formation in biosilification in metazoans’*? The sponge spicule is initially formed in the silicoblast in the form of a silica‐free “proteic rodlet”, which is produced in a great vacuoles. This axial rodlet was electron‐dense and of fibrillary nature, with spiral fibres 70–100 Å in diameter.^[^ ^20^ ^]^ The occurrence of these axial rodlets in the axial channels of spicules, not embedded in silica, has been observed previously using TEM (see for overview^[^ ^7^, ^18^ ^]^). The axial filament of F‐actin does not mineralize itself but rather provides the base for the mineralization around it. Moreover, while the distal tip of the spicule is open F‐actin can elongate, thus driving growth of the spicule. This may continue until the closure of the end of the spicule by mineralization, which stops spicule growth. This model of the spicule growth is supported by our observations and data. Actin's ability to bifurcate and to carry out dendrite branching at different angles is a well‐described.^[^ ^55^ ^]^ This plays a fundamental role in spiculo‐ and skeletonogenesis in sponges. We propose that the branching capability of actin filament bundles is an exaptation that led to the diverse skeletal forms of sponges, including 3D siliceous constructs at the macro‐level (Figures S4 and S11, Supporting Information).

The absence of hydroxyproline (3‐Hyp and 4‐Hyp) residues responsible for the attachment of silicic acid to the protein molecule, as is the case in collagen,^[^ ^14^ ^]^ further excludes actin as a direct template for silicification. It can, however, be a crucial template for other low (glassin, silicateins^[^ ^12^, ^13^, ^57^ ^]^), or high molecular weight biomolecules (hydroxylated collagen), which are major players in the biosilicification of sponges.^[^ ^7^ ^]^ Thus, actin appears in a new functional role as a driving force defining the diversity of sophisticated^[^ ^58^, ^59^ ^]^ biosilica architecture in sponges (Figure S3 and S4, Supporting Information). Our observations of actin‐rich axial filaments in diverse sponges using HRTEM show with strong evidence that they are cross‐linked and bundled together (Figure 4, and Figures S18–S21, Supporting Information). This feature makes such large‐scale actin constructs much stronger that an individual actin filament.

The conserved morphology of sponges through the fossil record suggests this is an ancient role that actin has played for at least 545 million years.^[^ ^60^ ^]^ We propose that F‐actin was localized in an ancestral, intracellular siliceous construct. As spiculogenesis and skeletogenesis moved to extracellular spaces, actin continued to play its pattern forming role, with the lack of an enclosing cell membrane releasing the size constraint on fibrous actin complexes.

The next challenge seems to lie in conducting experiments on the in vitro polymerization of actin monomers into filaments under model conditions in the presence of silicic acid. The prospect of using actin filaments to create new silicate‐containing materials and biomaterials with sophisticated three‐dimensional architecture is extremely interesting as a biomimetic model for mineralization under laboratory conditions.

## 4 Experimental Section

### Materials and Methods

*Sponge Species Used in the Study*: Representatives of glass sponges (class Hexactinellida): Aphrocallistes beatrix; Asconema setubalense; Caulophacus arcticus; Euplectella aspergillum; Farrea sp.; Hyalonema (Corynonema) populiferum; Malacosaccus sp., Monorhaphis chuni, Monorhaphis sp; Rossella antarctica; Waltheria flemmingii. Representatives of the class Demospongiae: Cladorhiza corona; Spongilla lacustris; Petrosia ficiformis; Geodia cydonium. The extraction of axial filaments from skeletal structures of sponges listed above by a sliding drop method is presented in the SI.

*Analytical Methods*: The SI also includes the analytical methods used in this work such as phalloidin staining, immunostaining, light, and fluorescent microscopy, scanning electron microscopy (SEM), transmission electron microscopy (TEM), high‐resolution transmission electron microscopy (HR‐TEM), fast Fourier transform (FFT), SDS‐PAGE and western blot, proteomic approach, mass spectrometry methods (LC‐MS/MS), and Raman spectroscopy. Experiments concerning influence of latrunculin B and germanic acid on spiculogenesis of living *S. lacustris* sponges are also described in detail there.

### Statistical Analysis

All the experiments were performed in triplicates and repeated at least three times (*n* = 3), unless otherwise stated. Statistical analyses were performed using GraphPad Prism software v. 5.01 (GraphPad Prism software Inc., La Jolla, CA, USA) using unpaired Student's t‐test for comparison of two groups. Data if necessary is presented as mean ± SD (standard deviation) (see Table S7, Supporting Information). The differences were considered to be statistically significant if p < 0.05 for either test used. Each Raman spectrum was acquired in 80 s or 200 s, i.e., a CCD integration time of 2 s was used in both measurements and several spectra (n = 40 with RamanRxn1 and n = 100 with Apha 300S) were averaged in order to improve the signal‐to‐noise ratio. The fluorescence background was removed with a multi‐point linear baseline using the software GRAMS/AI (Thermo Fisher Scientific, USA Inc, Waltham, MA, USA). Four different positions were measured on axial filaments extracted from glass sponges, in order check for homogeneity. All spectra acquired on each sample resulted totally similar; a representative one is displayed in Figure S23 (Supporting Information).

## Conflict of Interest

The authors declare no conflict of interest.

## Supporting information

Supporting information

## Data Availability

The data that support the findings of this study are available from the corresponding author upon reasonable request.

## References

[1] D. Pisani , W. Pett , M. Dohrmann , R. Feuda , O. Rota‐Stabelli , H. Philippe , N. Lartillot , G. Wörheide , Proc. Natl. Acad. Sci. USA 2015, 112, 15402. 26621703 10.1073/pnas.1518127112 PMC4687580

[2] M. J. Telford , L. L. Moroz , K. M. Halanych , Nature 2016, 529, 286. 26791714 10.1038/529286a

[3] P. Simion , H. Philippe , D. Baurain , M. Jager , D. J. Richter , A. Di Franco , B. Roure , N. Satoh , É. Quéinnec , A. Ereskovsky , P. Lapébie , E. Corre , F. Delsuc , N. King , G. Wörheide , M. Manuel , Curr. Biol. 2017, 27, 958. 28318975 10.1016/j.cub.2017.02.031

[4] M. Dohrmann , G. Wörheide , Sci. Rep. 2017, 7, 3599. 28620233 10.1038/s41598-017-03791-w PMC5472626

[5] Systema Porifera: A Guide to the Classification of Sponges (Eds: J. N. A. Hooper , R. W. M. van Soest ), Kluwer Academic/Plenum Publishers, Dordrecht 2002.

[6] R. W. M. van Soest , N. Boury‐Esnault , J. Vacelet , M. Dohrmann , D. Erpenbeck , N. J. De Voogd , N. Santodomingo , B. Vanhoorne , M. Kelly , J. N. A. Hooper , PLoS One 2012, 7, e35105. 22558119 10.1371/journal.pone.0035105 PMC3338747

[7] M. Wysokowski , T. Jesionowski , H. Ehrlich , Am. Mineral. 2018, 103, 665.

[8] A. Sola‐Rabada , M. Michaelis , D. J. Oliver , M. Roe , L. Colombi Ciacchi , H. Heinz , C. C. Perry , Langmuir 2018, 34, 8255. 29924624 10.1021/acs.langmuir.8b00874

[9] A. Pisera , M. Łukowiak , S. Masse , K. Tabachnick , J. Fromont , H. Ehrlich , M. Bertolino , Front. Zool. 2021, 18, 58. 34749755 10.1186/s12983-021-00440-x PMC8576975

[10] N. V. Povarova , N. A. Barinov , M. S. Baranov , N. M. Markina , A. M. Varizhuk , G. E. Pozmogova , D. V. Klinov , V. B. Kozhemyako , K. A. Lukyanov , Sci. Rep. 2018, 8, 16759. 30425281 10.1038/s41598-018-34965-9 PMC6233156

[11] J. N. Cha , K. Shimizu , Y. Zhou , S. C. Christiansen , B. F. Chmelka , G. D. Stucky , D. E. Morse , Proc. Natl. Acad. Sci. USA 1999, 96, 361. 9892638 10.1073/pnas.96.2.361 PMC15141

[12] K. Shimizu , J. Cha , G. D. Stucky , D. E. Morse , Proc. Natl. Acad. Sci. USA 1998, 95, 6234. 9600948 10.1073/pnas.95.11.6234 PMC27641

[13] K. Shimizu , T. Amano , R. Bari , J. C. Weaver , J. Arima , N. Mori , Proc. Natl. Acad. Sci. USA 2015, 112, 11449. 26261346 10.1073/pnas.1506968112 PMC4577155

[14] H. Ehrlich , R. Deutzmann , E. Brunner , E. Cappellini , H. Koon , C. Solazzo , Y. Yang , D. Ashford , J. Thomas‐Oates , M. Lubeck , C. Baessmann , Nat. Chem. 2010, 2, 1084. 21107374 10.1038/nchem.899

[15] H. Ehrlich , M. Krautter , T. Hanke , P. Simon , C. Knieb , S. Heinemann , H. Worch , J. Exp. Zool. 2007, 308B, 473. 10.1002/jez.b.21174 17520693

[16] H. Ehrlich , M. Maldonado , A. R. Parker , Y. N. Kulchin , J. Schilling , B. Koehler , U. Skrzypczak , P. Simon , H. M. Reiswig , M. V. Tsurkan , E. Brunner , Adv. Opt. Mater. 2016, 4, 1608.

[17] Thesaurus of Sponge Morphology in Smithsonian Contributions to Zoology (Eds: N. Boury‐Esnault , K. Rützler ), Smithsonian Institution Press, Washington DC 1997.

[18] M. J. Uriz , X. Turon , M. A. Becerro , G. Agell , Microsc. Res. Techniq. 2003, 62, 279. 10.1002/jemt.10395 14534903

[19] V. Schoeppler , E. Reich , J. Vacelet , M. Rosenthal , A. Pacureanu , A. Rack , P. Zaslansky , E. Zolotoyabko , I. Zlotnikov , Sci. Adv. 2017, 3, eaao2047. 29057327 10.1126/sciadv.aao2047 PMC5647122

[20] C. Levi , C. R. Acad. Sci. 1963, 256, 497.

[21] R. W. Drum , J. Ultrastruct. Res. 1968, 22, 12. 4297538 10.1016/s0022-5320(68)90046-4

[22] S. P. Leys , Biol. Bull. 1995, 188, 241. 29281331 10.2307/1542302

[23] G. D. Elliot , S. P. Leys , J. Exp. Biol. 2007, 210, 3736. 17951414 10.1242/jeb.003392

[24] J. Mitchell , S. A. Nichols , EvoDevo 2019, 10, 26. 31687123 10.1186/s13227-019-0139-0 PMC6820919

[25] J. Colgren , S. A. Nichols , A muscle‐related contractile tissue specified by myocardin‐related transcription factor activity in Porifera. bioRxiv. 2021, 10.1101/2021.04.11.439235.

[26] W. G. Palmer , J. Chem. Soc. 1930, 1656.

[27] R. E. Shore , Biol. Bull. 1972, 143, 689. 28368703 10.2307/1540191

[28] J. C. Weaver , L. I. Pietrasanta , N. Hedin , B. F. Chmelka , P. K. Hansma , D. E. Morse , J. Struct. Biol. 2003, 144, 271. 14643196 10.1016/j.jsb.2003.09.031

[29] C. Sabella , E. Faszewski , L Himic , K. M. Colpitts , J. Kaltenbach , M. M. Burger , X. Fernandez‐Busquets , J. Immun. 2007, 179, 5927. 17947666 10.4049/jimmunol.179.9.5927

[30] S. G. Greenberg , P. Davies , J. D. Schein , L. I. Binder , J. Biol. Chem. 1992, 2, 564. 1370450

[31] L. G. Frigeri , T. R. Radabaugh , P. A. Haynes , M. Hildebrand , Mol. Cell Proteomics 2006, 5, 182. 16207702 10.1074/mcp.M500174-MCP200

[32] Y.‐R. Lou , L. Kanninen , B. Kaehr , J. L. Townson , J. Niklander , R. Harjumäki , C. J. Brinker , M. Yliperttula , Sci. Rep. 2015, 5, 13635. 26323570 10.1038/srep13635 PMC4555166

[33] A. E. M. Adams , J. R. Pringle , Method Enzymol. 1991, 194, 729. 10.1016/0076-6879(91)94054-g 2005819

[34] E. Wulf , A. Deboben , F. A. Bautz , H. Faulstich , T. Wieland , Proc. Natl. Acad. Sci. USA 1979, 76, 4498. 291981 10.1073/pnas.76.9.4498 PMC411604

[35] R. Revilla‐I‐Domingo , C. Schmidt , C. Zifko , F. Raible , Genetics 2018, 210, 435. 30143594 10.1534/genetics.118.301121 PMC6216596

[36] K. Katoh , H. Ichikawa , H. Ishikawa , J. Electron. Microsc. 1991, 40, 70. 1907635

[37] D. Saczko‐Brack , E. Warchol , B. Rogez , M. Kröss , S. M. Heissler , J. R. Sellers , C. Batters , C. Veigel , Proc. Natl. Acad. Sci. USA 2016, 113, E8387. 27956608 10.1073/pnas.1612719113 PMC5206537

[38] L. T. Nguyen , L. S. Hirst , Phys. Rev. 2011, 83, 031910. 10.1103/PhysRevE.83.031910 21517528

[39] M. M. A. E. Claessens , C. Semmrich , L. Ramos , A. R. Bausch , Proc. Natl. Acad. Sci. USA 2008, 105, 8819. 18579789 10.1073/pnas.0711149105 PMC2449323

[40] T. Oda , H. Yanagisawa , Commun. Biol. 2020, 3, 585. 33067529 10.1038/s42003-020-01321-5 PMC7567829

[41] J. von der Ecken , M. Müller , W. Lehman , D. J. Mannstein , Nature 2015, 519, 114. 25470062 10.1038/nature14033 PMC4477711

[42] B. Tesson , M. Hildebrand , PlosOne 2010, 5, e14300. 10.1371/journal.pone.0014300 PMC3000822 21200414

[43] A. M. L. Van de Meene , J. D. Pickett‐Heaps , Eur. J. Phycol. 2004, 39, 93.

[44] A. M. M. Schmid , Nova Hedwiga 1980, 33,811.

[45] G. S. Blank , C. W. Sullivan , J. Phycol. 1983, 19, 39.

[46] S. A. Cohn , J. Nash , J. D. Pickett‐Heaps , Protoplasma 1989, 149, 130.

[47] G. M. Durak , C. Brownlee , G. L. Wheeler , Sci. Rep. 2017, 7, 15409. 29133928 10.1038/s41598-017-15562-8 PMC5684398

[48] K. R. Ayscough , J. Stryker , N. Pokala , M. Sanders , P. Crews , D. G. Drubin , J. Cell Biol. 1997, 137, 399. 9128251 10.1083/jcb.137.2.399 PMC2139767

[49] I. Spector , N. R. Shochet , Y. Kashman , A. Groweiss , Science 1983, 219, 493. 6681676 10.1126/science.6681676

[50] P. Ganot , E. Tambutté , N. Caminiti‐Segonds , G. Toullec , D. Allemand , S. Tambutté , eLife 2020, 9, e50022. 32039759 10.7554/eLife.50022 PMC7032929

[51] P. Novick , D. Botstein , Cell 1985, 40, 405. 3967297 10.1016/0092-8674(85)90154-0

[52] W. M. Morton , K. R. Ayscough , P. J. McLaughlin , Nat. Cell Biol. 2000, 2, 376. 10854330 10.1038/35014075

[53] J. Tyszka , U. Bickmeyer , M. Raitzsch , J. Bijma , K. Kaczmarek , A. Mewes , P. Topa , M. Janse , Proc. Natl. Acad. Sci. USA 2019, 116, 4111. 30782789 10.1073/pnas.1810394116 PMC6410838

[54] T. L. Simpson , M. Gil , R. Connes , J. P. Diaz , J. Paris , J. Morphol. 1985, 183, 117. 29969865 10.1002/jmor.1051830107

[55] V. I. Risca , E. B. Wang , O. Chaudhuri , J. J. Chia , P. L. Geissler , D. A. Fletcher , Proc. Natl. Acad. Sci. USA 2012, 109, 2913. 22308368 10.1073/pnas.1114292109 PMC3286980

[56] C. Eckert , H. C. Schröder , D. Brandt , S. Perovic‐Ottstadt , W. E. G. Müller , J. Histochem. Cytochem. 2006, 54, 1031. 16709731 10.1369/jhc.5A6903.2006

[57] S. Görlich , A. J. Samuel , R. J. Best , R. Seidel , J. Vacelet , F. K. Leonarski , T. Tomizaki , B. Rellinghaus , D. Pohl , I. Zlotnikov , Proc. Natl. Acad. Sci. USA 2020, 117, 31088. 33229574 10.1073/pnas.2019140117 PMC7733841

[58] J. Aizenberg , J. C. Weaver , M. S. Thanawala , V. C. Sundar , D. E. Morse , P. Fratzl , Science 2005, 309, 275. 16002612 10.1126/science.1112255

[59] G. Letort , A. Z. Politi , H. Ennomani , M. Théry , F. Nedelec , L. Blanchoin , PLoS Comput. Biol. 2015, 11, e1004245. 26016478 10.1371/journal.pcbi.1004245 PMC4446331

[60] J. Antcliffe , R. Callow , M. G. Brasier , Biol. Rev. 2014, 89, 972. 24779547 10.1111/brv.12090

