# Supplementary material for: Arrested in Glass: Actin within Sophisticated Architectures of Biosilica in Sponges
Source: Adv Sci (Weinh). 2022 Feb 13;9(11):2105059. doi: 10.1002/advs.202105059 (PMC9009123; doi:10.1002/advs.202105059)
Supplement: Supplementary file 1 — Supporting information [file ADVS-9-2105059-s001.pdf]

ADVANCED SCIENCE
Open Access

Supporting Information

for *Adv. Sci.*, DOI 10.1002/advs.202105059

Arrested in Glass: Actin within Sophisticated Architectures of Biosilica in Sponges

Hermann Ehrlich, Magdalena Luczak, Rustam Ziganshin, Ivan Mikšík, Marcin Wysokowski, Paul Simon, Irena Baranowska-Bosiacka, Patrycja Kupnicka, Alexander Ereskovsky, Roberta Galli, Sergey Dyshlovoy, Jonas Fischer, Konstantin R. Tabachnick, Iaroslav Petrenko, Teofil Jesionowski, Anna Lubkowska, Marek Figlerowicz, Viatcheslav N. Ivanenko and Adam P. Summers**

ADVANCED SCIENCE
Open Access

Supporting Information

for *Adv. Sci.,* DOI: 10.1002/advs.202105059

Arrested in glass: actin within sophisticated architectures of biosilica in sponges

Hermann Ehrlich, Magdalena Luczak, Rustam Ziganshin, Ivan Miksik, Marcin Wysokowski, Paul Simon, Irena Baranowska-Bosiacka, Patrycja Kupnicka, Alexander Ereskovsky, Roberta Galli, Sergey Dyshlovoy, Jonas Fischer, Konstantin R. Tabachnick, Iaroslav Petrenko, Teofil Jesionowski, Anna Lubkowska, Marek Figlerowicz, Viatcheslav N. Ivanenko, Adam P. Summers**

Supporting Information

Arrested in glass: actin within sophisticated architectures of biosilica in sponges

Hermann Ehrlich, Magdalena Luczak, Rustam Ziganshin, Ivan Miksik, Marcin Wysokowski, Paul Simon, Irena Baranowska-Bosiacka, Patrycja Kupnicka, Alexander Ereskovsky, Roberta Galli, Sergey Dyshlovoy, Jonas Fischer, Konstantin R. Tabachnick, Iaroslav Petrenko, Teofil Jesionowski, Anna Lubkowska, Marek Figlerowicz, Viatcheslav N. Ivanenko, Adam P. Summers**

Supplementary Text

Note 1. List of sponge specimens with geographical coordinates of collection places: Hexactinellida

Aphrocallistes beatrix Aphrocallistidae: RV 'Academik Mstyslav Keldish' – 28, trawl, sta. 2750, 59° 50-47.5' N 29° 42-45' W, depth 1024-667 m.

Asconema setubalense Rossellidae: RV 'Vityaz 2' - 2, trawl, sta. 75, 36° 43.01' N, 14° 13.6' W, depth 280–300 m.

Caulophacus arcticus Rossellidae: RV 'Academik Mstyslav Keldish' – 40, submercible 'MIR 1', sta. 3786, 76° 46,77' N 7° 21.7 E, depth 3617-3410 m.

Euplectella suberea Euplectellidae: RV 'Academik Mstyslav Keldish' – 43, submercible 'MIR 1', sta. 3988, 44° 57,4' N 28° 0.9' W, depth 2800 m.

Farrea sp. – *Farrea* sp.n. Farreidae : RV 'Academik MA Lavrentyev' – 86, ROV 'Comanche', sta. 12, 38.7811° N 171.0917° E, depth 2152-1579 m.

Hyalonema (Corynonema) populiferum Hyalonematidae - 44°37'39.5"N 124°04'13.4"W, depth 900 m

Hyalonema sp. Hyalonematidae collected from a depth of 5,000 m in the Pacific (12 8N, 137 8E)

Malacosaccus sp. Euplectellidae: RV 'Academik Kurchatov' – 11, trawl, sta. 914, 56° 21-20' S 50° 48-47' W, depth 5700-5650 m.

Monorhaphis chuni Monorhaphididae and *Monorhaphis* sp. Monorhaphididae: RV 'Vityaz 2' - 17, trawl, sta. 2601, 12° 31.50-25.04' S, 48° 05.50–08.00' E, depth 700 m.

Rossella antarctica Rossellidae: RV 'Skif' – 3, trawl 169, sta. 1161/250, 45° 46,9' S 49° 59.1' E, depth 252-276 m.

Walteria flemmingi Euplectellidae - RV 'Academik MA Lavrentyev' – 86, ROV 'Comanche', sta. 13, 38.7665° N 171.0988° E, depth 1290-862 m.

Demospongiae

Cladorhiza corona Cladorhizidae: RV 'Academik MA Lavrentyev' – 75, ROV 'Comanche', sta. 16, 55.5774° N 167.3258° E, depth 4277-4278 m

Spongilla lacustris Spongillidae – Lake Riesenstein, Meißen, Germany - 51°10'01.9"-13°29'43.5"E, depth 2 m.

Petrosia ficiformis Petrosiidae Marseille, France; cave Endoume 43°16'47,45''N - 5°21'05,16E

Geodia cydonium Geodiidae Marseille, France, Cave Coral, Maire island 43°12'37,60" N - 5°20'24,86'' E

Note 2. Isolation of the axial filaments

a) **for Phalloidin- and immunostaining**

Selected spicules (n=45) and skeletal frameworks (n=25) (see **Figure 1** and **Figures S5-S11**, Supporting Information) have been treated in 70% HNO_3 at room temperature (RT) up to 5 times during 72 h for elimination of possible external organic layers. The absence of residual organic matter has been confirmed using fluorescence microscopy as well as Coomassie blue R-250 staining for proteins as well as using X-ray photoelectron spectroscopy (XPS). Organic-free skeletal structures have been rinsed in dist. H_2O up to pH 6.5, dried on air at RT and placed on the Nunc™ Permanox™ (Thermo Fisher Scientific, USA) plastic microscope slides (27/75mm) (n=120) in small drops of water. After water evaporation, one drop of 10% HF acid as recognized silica demineralizing reagent was applied to the surface of the spicules, or corresponding fragment of the skeletal framework. In order to prevent HF vapour from entering the environment, the slide has been placed at RT, inside the Plexiglas Petri dish at 10° angle. This positioning of the sample allows the HF-droplet to slowly roll down the surface of the plastic slide and removal of the dissolved silica, releasing the axial filaments during 7-10 h (see **Figure S1a**, Supporting Information). Following such treatment, the residual axial filaments remain to be strongly fixed to the plastic surface and have been rinsed with water during 2 h, dried on the air, and after that stained with Phalloidin reagents, or with fluorescently labelled ß-actin antibodies (see **Note 3,** Supporting Information).

It is to note that HF acid should not destroy the peptide bonds and that proteins treated with it display the same biochemical properties as normal. In work by Lou *et al.* [61], it was shown that silica bioreplication preserves three-dimensional spheroid structures of human pluripotent stem cells and HepG2 cells. The authors used diluted in buffer hydrofluoric acid for remove silica composites, and obtained spheroid was successfully stained for F-actin using antigens staining.

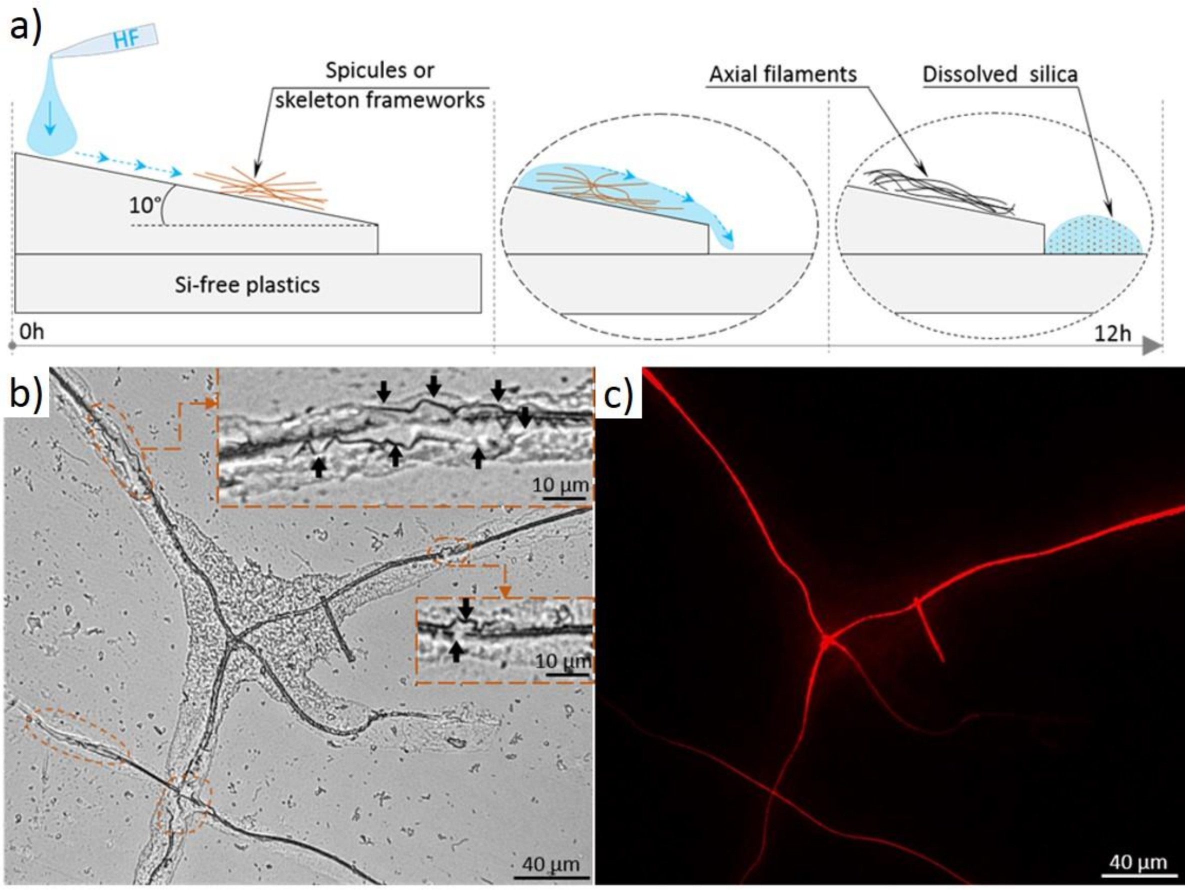

Figure S1. (a) Schematic diagram depicting the extraction of axial filaments from skeletal structures of sponges by a sliding drop method. The effectiveness of this method never approved before is visualized on the images (**b**) and (**c**). Light microscopy image (**b**) represent the case of partially demineralized skeletal framework of *Euplectella* sp. glass sponge after staining with iFluor 594-Phalloidin, where the residual silica is signed with dotted lines and visualized in the insertion with arrows under higher magnification. The fluorescence image (**c**) of the same sample shows with strong evidence localization of actin-based axial filaments even being located with residual silica. It should be noted that the remains of the organic matrix, clearly visible in the image (**b**) in the form of a case-like structure, were not stained with highly specific to actin phalloidin dye, in contrast to the axial filaments (**c**).

b) for Immunostaining, MS, Raman, SDS-PAGE

Organic-free skeletal structures of selected sponges under study have been prepared as described above, rinsed in dist. H_2O up to pH 6.5, dried on air at RT and, then placed in 50 ml plastic vessels containing 25 ml of 10%HF (Fluka) solution according to the method by Drum [21]. The vessel was covered, placed under thermostatic conditions at 25°C for time periods between 24 h and 96 h in dependence of the sponge species. Extracts obtained after dissolution of biosilica were dialyzed against deionized water (10 L) on Roth (Germany) membranes with a molecular weight cut-off 14 kDa at 4°C during 72 h. The dialyzed material

was stored at 4°C and used for actin identification as described in detail in the notes 5 and 6, Supporting Information.

Note 3. Staining with Phalloidins and Immunostaining

Axial filaments were stained with the actin staining kits: Cell Navigator™ F-Actin Labeling Kit *Red Fluorescence*; Cell Navigator™ F-Actin Labeling Kit *Green Fluorescence*; Cell Navigator™ F-Actin Labeling Kit *Blue Fluorescence* provided by AAT Bioquest (USA). iFluor 594-Phalloidin (red); iFluor™ 488-Phalloidin (green) and iFluor™ 350-Phalloidin (blue) fluorescent phalloidin conjugates are a high-affinity probes for F-actins. Used at nanomolar concentrations, phallotoxins are convenient probes for labelling, identifying and quantitating especially of F-actins. In brief, isolated axial filaments fixed on the Nunc™ Permanox™ (Thermo Fisher Scientific) plastic microscope slides were treated with 100 µL/well of iFluor™ 594-Phalloidin or iFluor™ 488-Phalloidin or iFluor™ 350-Phalloidin working solution and stained at RT. Afterwards the plates were carefully washed 5 times with dist. water over 1 h, dried and observed using light and fluorescent microscopy.

For immunostaining, the samples of both partially demineralized and mineral-free isolated axial filaments were placed on the sample glasses in 30 µL of dH_2O and were dried overnight. Then 30 µL of 4% PFA/PBS were added over each sample and the samples were incubated for 20 min in the wet chamber. The PFA solution was removed and the samples were washed with PBS (3 x 5 min, 30 µL/sample). The samples were blocked with 30 µL of 2% of normal goat serum in PBS were added over each sample and the samples were incubated for 30 min in the wet chamber. The primary anti-β-actin antibody (#PA1-16889, Invitrogen) were diluted 1:200 in the buffer containing 3% BSA / 0.02% NaN_3 / PBS according to the manufacture’s protocol. 30 µL of the primary antibody solution were placed over each sample and the glasses were incubated overnight in the wet chamber at +4°C. Then the samples were washed with PBS (3 x 5 min, 30 µL/sample). The secondary antibody (Anti-rabbit IgG (H+L), F(ab')2 Fragment (Alexa Fluor® 488 Conjugate) #4412, Sell Signaling Technology) were diluted in PBS according to the manufacture’s protocol and 30 µL of this solution were placed over each sample and the glasses were incubated for 1 h in the wet chamber at room temperature. Then the samples were washed with PBS (3 x 5 min, 30 µL/sample). The DAPI-free mounting medium was added over each sample and the the glass slides were covered with glass slips and immediately analyzed using fluorescent AxioScope.A1 (Carl Zeiss, Göttingen,

Germany) microscope and with the AxioVision40 V4.8 software (Carl Zeiss Imaging Solutions, Göttingen, Germany).

The samples of the axial filaments under study, which have been treated only with secondary antibodies (#4412, Sell Signaling Technology) have been used as control.

As you can see, there is principal distinction with respect to reagents (i.e. PFA/PBS treatment) and reaction conditions between phalloidin-based staining and immunostaining of axial filaments under study. Due to fundamental differences with regard to the handling of samples, we did not expect the same image after visualization with respect to thickness and density.

Note 4. Stereo, light and fluorescent microscopy

Stereomicroscopy images were taken with a Keyence VHX-5000 digital optical microscope and VH-ZST swing-head zoom lens. Light microscopy and fluorescent images were obtained using a Keyence BZ-9000 fluorescence microscope.

Note 5. SDS-PAGE and western blotting

Axial filament samples isolated from *A. setubalense*, *S. lacustris* and *C. arcticus* were precipitated with ice cold acetone, incubated at -20°C and centrifuged at 10,000 g for 10'. Pellets were resolved in 2% SDS (*A. setubalense*), in SDS lysis buffer (*S. lacustris;* 0.1% SDS, 1% NP-40, 50 mM Tris-HCl (pH 7.6), 0.88% NaCl, 0.25% sodium cholate, 1 mM Na_3VO_4, 0.1 mM PMSF, protease inhibitor cocktail cOmplete mini EDTA-free (Roche, Germany)) or in 2x Laemmli buffer/SDS lysis buffer (1:1; *C. arcticus*). The samples were sonicated, vortexed for 3 h and centrifuged at 10,000 g for 10'. Protein concentration was measured using a commercial BCA kit (Pierce) or Bradford assay. 18-20 μg of total protein was separated on gradient 4-15% SDS-PAGE (Biorad, USA), fixed and stained with colloidal Coomassie Blue-G250 overnight, followed by destaining with water.

For western blot analysis, proteins were transferred to PVDF membrane using the Trans-Blot^{TM} Turbo system (Bio-Rad, USA) and blocked with TBS-T/4 - 5% BSA. Blots were incubated overnight with the IgG anti- ß- actin (for *S. lacustris* #5125, Cell Singling, Beverly, MA, USA; for *A. setubalense* - #PA1-16889, Thermo Fisher Scientific, USA) as a primary antibody. Chemiluminescent detection was performed using ChemiDoc XRS imaging system (Biorad, USA) or ECL chemiluminescence system (Thermo Fisher Scientific, USA) according to the manufacturer protocol.

Since there is no information on the sequence of actin from *S. lacustris* in the Uniprot database, we do not know what molecular mass the actin from this sponge should have. We suggest that actin in this sponge species has a peptide bond that is labile under the conditions of dissolution of spicules, the cleavage of which divides the molecule into two unequal parts: 25 and 15 kDa; protein bands corresponding to precisely these molecular weights are observed in the Western blotting picture (**Figure S15a, b,** Supporting Information).

Note 6. LC-MS/MS analysis

To identify proteins separated using SDS-PAGE, bands were manually excised from gels and transferred to Eppendorf tubes. Each gel piece was washed twice with 100 µl of 25 mM ammonium bicarbonate (AMBIC), 50% ACN and dehydrated for 15 min by addition of 100 µl of ACN, followed by 45 min incubation in 10 mM DTT/50 mM AMBIC at 56 °C. The solution was then replaced by 55 mM iodoacetamide/50 mM AMBIC for 30 min in the dark, at RT. After removal of the iodoacetamide solution, a 15 min washing step in 100 µl of 25 mM AMBIC and 50% ACN was performed. Following drying under vacuum, the gel pieces were rehydrated with 10 µl of 20 µg/ml of modified trypsin (Promega (USA), sequencing grade) in 50 mM AMBIC and overnight digested at 37 °C. Digestion was stopped by acidification with 1 µl of TFA. The gel pieces were subsequently sonicated for 10 min to elute peptides, and obtained mixtures were analysed by LC-MS/MS in order to protein identification.

Axial filaments samples were also directly analysed by LC-MS/MS (without a prior SDS-PAGE separation) to identify proteins present in extracts. For this purpose, in-solution digestion and label-free LC-MS/MS approaches were utilized. Actin samples were precipitated with acetone as described for SDS-PAGE analysis. For *A. setubalense* samples, the pellet was resolubilized in 200 mM AMBIC overnight with shaking, followed by heating at 70 °C (2 x15') and 5 times sonication step. Protein concentration measurements were subsequently applied. For *M. chuni* and *C. arcticus* axial filaments, samples were resolved in 100 mM Tris pH 8.5, 10 mM TCEP, 20 mM 2-chloroacetamide and 1% sodium deoxycholate (SDC), sonicated 5'using ultrasonic water bath, heated at 95°C for 10', and diluted 2-times with 100 mM Tris (pH 8.5). Samples were digested with trypsin at 37°C overnight and then reaction was stopped with 0.1% TFA. The peptides were analysed by LC-MS/MS.

For protein identification, LC-MS/MS analyses were performed using three different systems:

a) Ultimate 3000 NanoLC coupled to Q-Exactive Orbitrap mass spectrometer (Thermo Fisher Scientific). Analyses were performed using an RP C18 precolumn (Thermo Fisher Scientific, USA) connected to a 75 um i.d. x 25 cm RP C18 Acclaim PepMap column with a particle size of 2 µm and a pore size of 100 Å (Thermo Fisher Scientific, USA). The following LC buffers were used: buffer A (0.1% (v/v) formic acid in Milli-Q water) and buffer B (0.1% formic acid in 90% acetonitrile). The peptides were eluted from the column in 185 min method with a constant flow rate of 300 nl/min and a linear gradient of buffer B (5%-65%). Q-Exactive Orbitrap settings were as follows: the resolution was set to 70,000 for MS scans, and 17,500 for the MS/MS scans. The MS scan range was from 300 to 2000 *m/z*. The isolation window was set to 2 *m/z*.

Following LC-MS/MS analysis, the raw files were analysed by Proteome Discoverer, version 2.2 (Thermo Fisher Scientific, USA). The identification of proteins was performed using the SEQUEST engine against the UniProtKB/TrEMBL database for Porifera (49,423 sequences) and UniProtKB/Swiss-Prot reviewed database with all entries (469,134 sequences) using the following parameters: a tolerance level of 10 ppm for MS and 0.08 Da for MS/MS. Trypsin was selected as the digesting enzyme with 1 missed cleavage allowed. The carbamidomethylation of cysteine was set as a fixed modification, and the oxidation of methionine as variable modification.

b) Ultimate 3000 NanoLC coupled to Q-Exactive HF Orbitrap mass spectrometer (Thermo Fisher Scientific, USA). Analyses were performed using a trapping column (100 µm internal diameter, 20 mm length, packed in-house with Aeris Peptide XB-C18 2.6 µm resin (Phenomenex) connected to 25-cm 75 µm inner diameter column packed in-house with Aeris Peptide XB-C18 2.6 µm resin (Phenomenex). The following LC buffers were used: buffer A (0.1% (v/v) formic acid in Milli-Q water) and buffer B (0.1% formic acid in 80% acetonitrile). The peptides were eluted from the column with a constant flow rate of 350 nl/min with a linear gradient of buffer B from 5% to 45%. Q-Exactive HF Orbitrap settings were as follows: the resolution was set to 60,000 for MS scans at 200 m/z, and 15,000 for the MS/MS scans at fixed first mass 100 *m/z*. The MS scan range was from 300 to 1,400 m/z. The isolation window was set to 1.2 *m/z*.

Data analysis has been carried out using Peaks Studio 8.0 software. Peptide list generated by Peaks studio was searched against various databases including UniProt/TrEMBL database of Metazoa (with 11,402,693 sequences). Cysteine carbamidomethylation was set as a fixed

modification, methionine oxidation and asparagine and glutamine deamidation as variable modifications. Enzyme specificity was set to trypsin with D|P. Peptide identification was performed with a tolerance level of 10 ppm for MS and 0.05 Da for MS/MS. False discovery rate (FDR) was set to 0.01 for peptide-spectrum matches and determined by performing a reverse database search.

c) nano-HPLC system Proxeon Easy-nLC (Proxeon, Odense, Denmark) coupled to MaXis Q-TOF mass spectrometer (Bruker Daltonics, Bremen, Germany). Analyses were performed using an NS-AC-12dp3-C18 Biosphere C18 column (particle size: 3 µm, pore size: 12 nm, length: 200 mm, inner diameter: 75 µm) with an NS-MP-10 Biosphere C18 precolumn (particle size: 5 µm, pore size: 12 nm, length: 20 mm, inner diameter: 100 µm), both manufactured by NanoSeparations (Nieuwkoop, Holland). The following LC buffers were used: buffer A (0.1% (v/v) formic acid in Milli-Q water) and buffer B (0.1% formic acid in acetonitrile). The peptides were eluted from the column with a constant flow rate of 200 nl/min with a step gradient of buffer B from 5% to 50%. MaXis Q-TOF settings were as follows: the ESI voltage was set to +4.5 kV, scan time: 3 Hz; drying gas (N_2): 4 L/min; drying gas temperature: 180 °C; nebulizer pressure: 100 kPa. The MS scan range was from 50 to 2,000 *m/z*.

Data were processed using ProteinScape software v. 3.0.0.446 (Bruker Daltonics, Germany). Proteins were identified by correlating tandem mass spectra to the extracted database for sponges from the NCBI database (downloaded on 27th February 2018; 33,624 sequences; 13,814,996 residues), using the MASCOT searching engine v. 2.3.0. Trypsin was set as the digesting enzyme. Two missed cleavages were allowed with a tolerance level of 15 ppm for MS and 0.03 Da for MS/MS. Variable modifications were set as: proline and lysine were allowed to be hydroxylated, whereas asparagine and glutamine deamidated. The Peptide Decoy option was selected during the data search process to remove false-positive results. Only significant hits were accepted (MASCOT score ≥80 for proteins and MASCOT score ≥20 for peptides.

As a result, 4 and 7 actin peptides were identified for *A. setubalense* analysed by SDS-PAGE/LC-MS/MS or label-free LC-MS/MS approach, respectively (**Figure 3, Table 1,** Supporting Information). Three actin peptides also identified in *S. lacustris* samples separated by SDS-PAGE (**Figure S15**). In these samples 4 silicatein peptides were also found.

Characteristics of these peptides and representative spectra are presented in Supporting Information, **Table 5** and **Figure S16** and **S17**.

In the case of *M. chuni, M. sp and C. arcticus* 14, 15 and 21 actin peptides were identified utilizing label-free LC-MS/MS approach without SDS-PAGE separation, respectively. Sequences and results of these analyses are presented in Supporting Information, **Tables 2, 3** and **4**. Representative spectra are presented in **Figure S16**, Supporting Information.

Note 7. Scanning electron microscopy

The samples were fixed in a sample holder and covered with carbon, or with a gold layer for 1 min using an Edwards S150B sputter coater. The samples were then placed in an ESEM XL 30 Philips or LEO DSM 982 Gemini scanning electron microscope.

Note 8. Ultramicrotomy and transmission electron microscopy

Dry axial filaments of glass sponges, *C. arcticus* and *M. chuni* were placed in ethanol 30% for one day at RT. They were subsequently dehydrated in ethanol series at RT and stored in 100% ethanol at 4°C. Then they were cut into short fragments using micro scissors. The samples were embedded in Araldite (Sigma-Aldrich) epoxy embedding media according to the manufacturer instructions. Ultrathin sections (60–80 nm) were cut with an Ultramicrotome PowerTome XL, equipped with a Drukkert 45° diamond knife, and contrasted with UranyLess (EMS) solution. Ultrathin sections were studied under JEOL JEM 1400 transmission electron microscope.

Experiment with Latrunculin B inhibition. Selected samples, 5 mm in diameter, of young *S. lacustris* sponges were fixed for 5 h in 2.5% glutaraldehyde in 0.1 M sodium cacodylate buffer (osmolarity adjusted to 980 mOsM with saccharose) at 4°C, washed three times in the buffer solution, postfixed for 90 min in 2% osmium tetroxide (OsO4) in the same buffer, dehydrated in an acetone series and embedded as decribed above. Ultrathin sections have been contrasted with uranyl acetate and lead citrate.

Note 9. High-resolution transmission electron microscopy (HRTEM)

TEM analyses were performed using a FEI Tecnai F30-G2 with Super-Twin lens (Thermo Fisher, Eindhoven, NL) with a field emission gun at an acceleration voltage of 300 kV. The point resolution amounts to 2.0 Å, and the information limit to about 1.2 Å. The microscope is

equipped with a wide-angle slow scan CCD camera (MultiScan, 2k×2k pixels; Gatan Inc., Pleasanton, CA, USA).

Note 10. Raman spectroscopy

Raman spectroscopy was performed on extracted axial filaments and on different reference proteins. For instance, actin from rabbit muscle (p/n A2522, Sigma Aldrich, Taufkirchen, Germany), bovine albumin (p/n 11930, SERVA Electrophoresis GmbH, Heidelberg, Germany), human collagen type I (p/n C7774, Sigma Aldrich, Taufkirchen, Germany), gelatin from cold water fish skin (G7765, Sigma Aldrich), silicatein A1 from *Latrunculia oparinae* demosponge (TIBOH, Vladivostok); papain from papaya latex (P3375, Sigma Aldrich), pepsin from porcine gastric mucosa (p/n 1.07192, Sigma Aldrich) and trypsin from porcine pancreas (p/n T4799, Sigma Aldrich) were used.

Raman spectra of the reference proteins and of axial filaments extracted from glass sponges *M. chuni* and from *C. arcticus* were recorded with a Raman spectrometer (RamanRxn1™, Kaiser Optical Systems Inc., Ann Arbor, USA) coupled to an upright microscope (DM2500 P, Leica Microsystems GmbH, Wetzlar, Germany). The excitation of Raman scattering was obtained with a diode laser with wavelength 785 nm, which was propagated to the microscope with a 62.5 µm optical fiber and focused on the samples by means of a 20x/0.45 microscope objective, leading to a focal spot of about 35 µm. The Raman signal was collected in reflection configuration and propagated to the spectrograph using an optical fiber with 100 µm core. The spectral resolution is 4 cm^{-1}. Raman spectra were punctually recorded, using a laser power of about 150 mW. Raman spectra of single axial filaments isolated from *Asconema setubalense* and *Petrosia fuciformis* placed on glass substrates were acquired using a confocal Raman microscope (Alpha 300S, WITec GmbH, Ulm, Germany) coupled to a Raman spectrometer UHTS 300S) and using a laser excitation at 780 nm with TEM00 quality (TA Pro, Toptica Photonics AG, Gräfelfing, Germany). A 100x magnification objective with NA=0.9 was used to focalize the excitation and collect the Raman signal in reflection configuration. Raman spectra were punctually recorded on single fibres, using a laser power of about 30 mW.

An integration time of 2 s was used in both measurements and several spectra were averaged in order to improve the signal-to-noise ratio. The fluorescence background was removed with a multi-point linear baseline using the software GRAMS/AI (Thermo Fisher Scientific, USA Inc, Waltham, MA, USA).

Note 11. Latrunculin B inhibition

Gemmules isolated from *S. lacustris* were carefully placed on the sterile glass slides. The hatching of the gemmules was performed by placing them on glass slides in 60 ml of tap water in plastic Petri dishes at RT. During the whole experiment all slides were constantly covered with water, and all containers were covered to prevent the loss of water through the evaporation. First hatching was observed after 72h. The microscope slides containing one or more hatched gemmules were prepared and used for actin inhibition experiments (**Figure 5** and **Figure S24**, Supporting Information). To analyse the impact of actin inhibition, slide with gemmules was treated with 2 ml of tap water containing 1 µM of latrunculin B (purity ≥98%; Cat. No. 3974, Tocris, USA), dissolved in dimethyl sulphoxide (DMSO), for 8 days. Each day the culture media was replaced with a fresh one. As a reference, corresponding slides with hatched gemmules were treated under similar experimental conditions with 2 ml of tap water, and 2 ml of tap water mixed with 10 µl of DMSO. The impact of actin inhibitor on sponge growth and spiculogenesis was assessed every 24 h by the light (Keyence BZ 9000, Osaka, Japan), as well as scanning electron microscopy (**Figure 5**).

In total of 72 used gemmules, 64 could be observed to hatch. Of the 22 hatched gemmules treated with the inhibitor, none one has been observed to grow with spicules. All hatched gemmules, treated with DMSO or only tap water respectively, were observed to contain spicules during the growing phase.

Latrunculin B, actually originally discovered in sponges, inhibits actin polymerization. This Latrunculin is highly specific and does not kill or stop cell function. Cell viability during and after treatment with Latrunculin is well known and recently has, for example, been confirmed by Durak, et al. [47] and by by Ayscough et al [62]. The evidences that the inhibition of F-actin by Latrunculin is reversible has been shown in vertebrate cultured cells [63] and in corals [64].

In our experiments with Latrunculin we used protocols worked out on other aquatic organisms. This informed our selection of concentration and duration of exposure, so they did not affect the physiology of individual cells or the viability of the whole organism. The duration of the experiments was due to the well-studied feature of the development of *S. lacustris* sponges after their release from the gemmules and the time required for the development of spicules under normal conditions. Recovery experiments were not necessary since cells were observed to be live and not lysed daily via light microscopy, also visible in SEM (**Figure 5**).

Note 12. Germanium experiment

We have used the same gemmules from *S. lacustris* as described in the **note 11, Supporting Information**. To analyse the influence of germanium (Ge) on the morphology of axial filaments in control experiment, slides (n=17) with gemmules were treated with 2 ml of tap water each containing 0.35 mM of Na_2SiO_3 (Sigma-Aldrich). For the comparative experiment, sufficient quantities of GeO_2 (Sigma-Aldrich) were added to yield Ge/Si molar ratios of 0.5. Each day these two-culture media were correspondingly replaced with fresh one. The impact of germanium on sponge spiculogenesis was assessed every 24 h by the light and fluorescence microscopy (Keyence BZ 9000, Osaka, Japan) within a course of 8 days.

Note 13. Axial filaments and sclerocytes

Sclerocytes are recognized as cells, in which the siliceos spicules appear. The cytoplasm of an active sclerocyte is filled with abundant small clear vesicles, mitochondria and numerous microtubes [65-57].The spicules are secreted intracellularly within a special vacuole, the membrane of which was termed a silicalemma. The silicalemma is different from the cell membrane [66]. The silicalemma is often very close to the plasma membrane, but no obvious connections between the plasma membrane and silicalemma have been observed. As suggested by Simpson [68], the silicalemma seems to be an invagination (in pocketing) of the cell membrane and therefore, the axial filament and silica occur in an elongate, extracellular pocket. Therefore, silicalemma appears to form an elongate vacuole in the cytoplasm with no direct connections to the Golgi complex, endoplasmic reticulum, or other membrane system. Spiculogenesis begins with the synthesis of the axial filament, which is then mineralized by silica deposition, however, in such a way that the space around it remains free of mineral phase (see **Figure S2,** Supporting Information). The deposition of silica is due to the secretory activity of the silicalemma, which enlarges as additional silica is deposited. Thus, the silica closest to the axial filament is the oldest (denser) and that at the periphery is youngest (least dense) [68].

After the formation of the spicule, the sclerocyte either slides off the newly formed megascleres, or it is transported into the extracellular space. Sclerocyte cells at the end of spiculogenesis do not undergo apoptosis or death. The same cell can start the synthesis of a next spicule [69]. A micro tubular material, more or less organized, is often seen surrounding the axial filament at the first stages of spicule development [70]. It is not known whether these microtubules are present only during the early assembly of the filament. All the current evidence indicates that the axial filament is formed by active sclerocytes during the early

stages of spiculogenesis. Axial filament remains an active and important participant in the development of spicules. The axial filament may have as its major function the establishment of the overall morphology of the developing spicule both microscleres and megascleres. Up to now there is only one hypothesis of axial filament origin, proposed by Lévi [71], further elaborated by Simpson [68]: axial filament forms from the fusion of cytoplasmic vacuoles, developed by invagination of the cell membrane.

Pottu-Boumendil [53] and Simpson et al. [50] described the appearance of hexagonal axial filament in demosponges, surrounding with microtubules in the sclerocyte cytoplasm at a very early stage of spiculogenesis. According to their TEM micrographs, immediately after this, the axial filament is surrounded by a layer of silicalemma adjacent to its surface before development of a vacuole. This observation allows us to propose the second hypothesis of the formation of axial filament in sclerocytes of sponges, namely, its development due to the condensation of extracellular F-actin nano-fibrils.

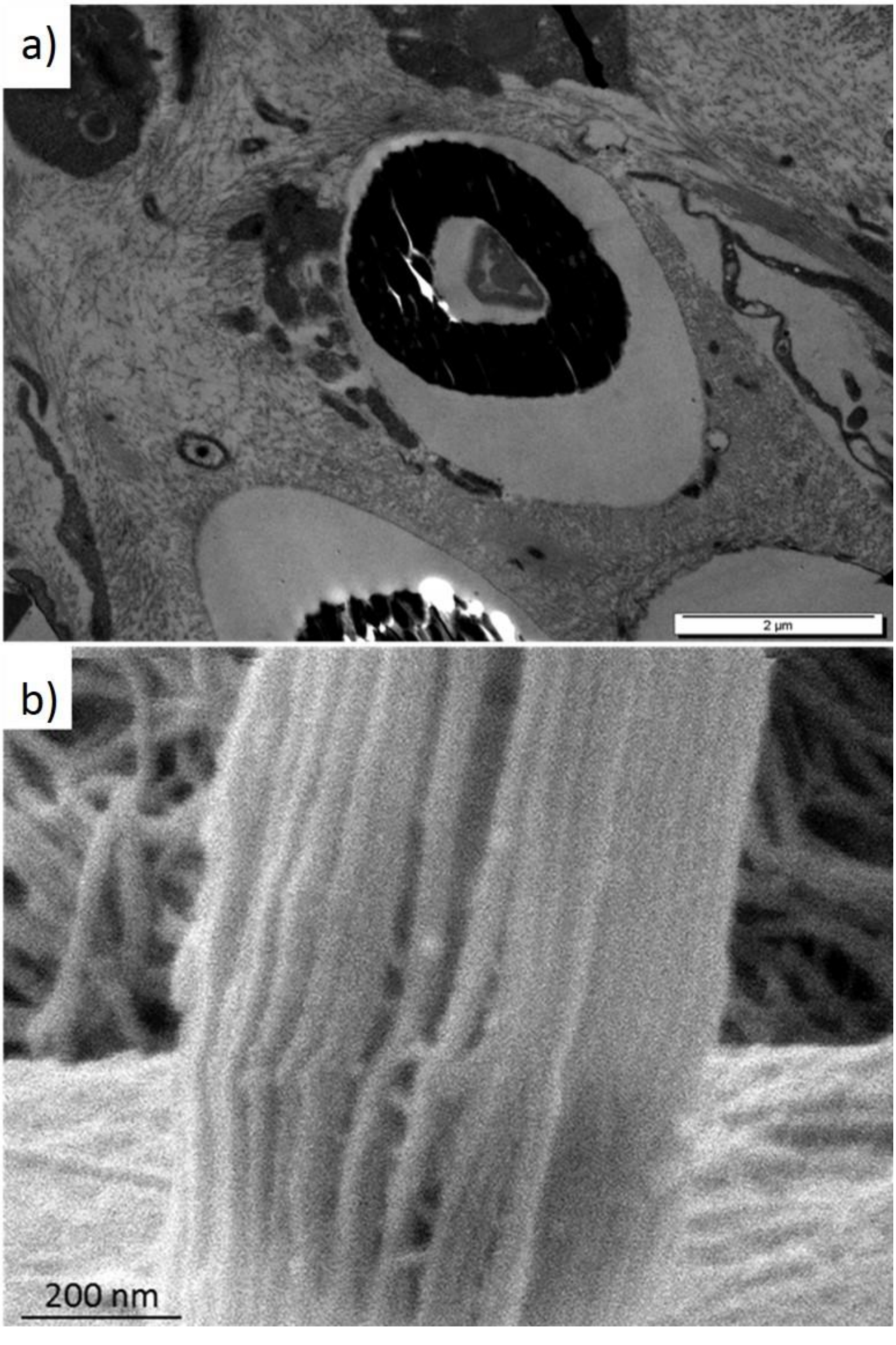

Figure S2. Localization of axial filament within young and adult spicules. (**a**)TEM image of the cross-section through the sclerocyte of *S. domuncula* demosponge and the siliceous spicule (black colour) together with triangular axial filament in the middle of spicule (electron dense matter). It is clearly visible that the axial filament is not closely embedded into silica, but lays free within it. (Image courtesy of Prof. A. Ereskovsky). (**b**) The axial filament remains non-mineralized in the finally formed spicule as represented on the SEM image. The fibrous filament turned out to be visible as a result of a mechanical fracture of a spicule

isolated not from the museum's, but from freshly collected material. Cross-links between individual nanofibers are also well visible.

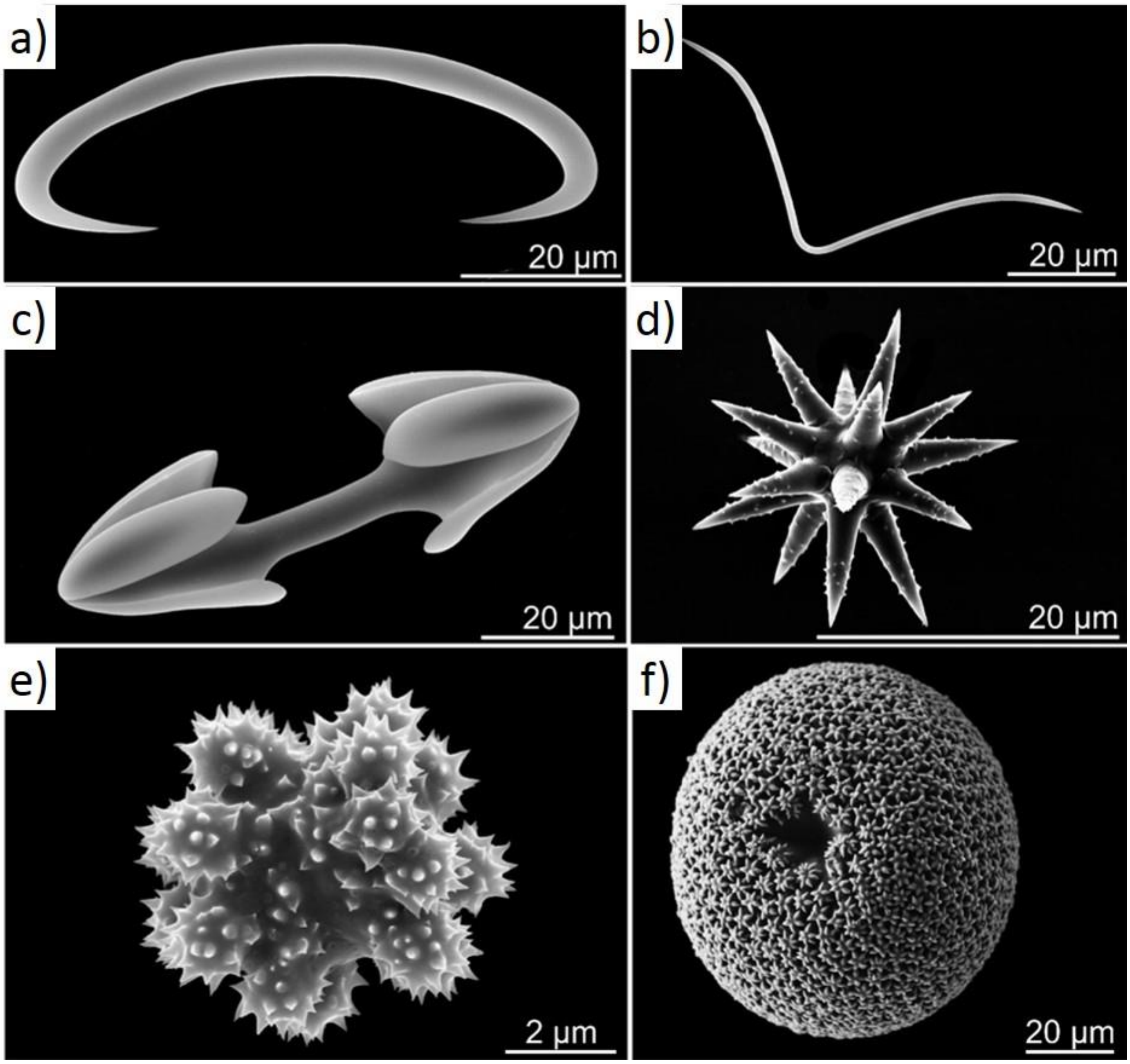

Figure S3. SEM images of the selected siliceous microscleres of Demospongiae**.** (**a**) sigma of *Abyssocladia koltuni*; (**b**) toxa of *Microciona armata*; (**c**) anchorate spatuliferous isochela of *Abyssocladia koltuni*; (**d**) oxyaster of *Geodia atlantica*; (**e**) strongylaster of *Geodia barretti*; (**f**) sterraster of *Geodia atlantica*. (**a** - **c** - photos of A. Ereskovsky; **d** - **f** – photos of courtesy of P. Cardenas).

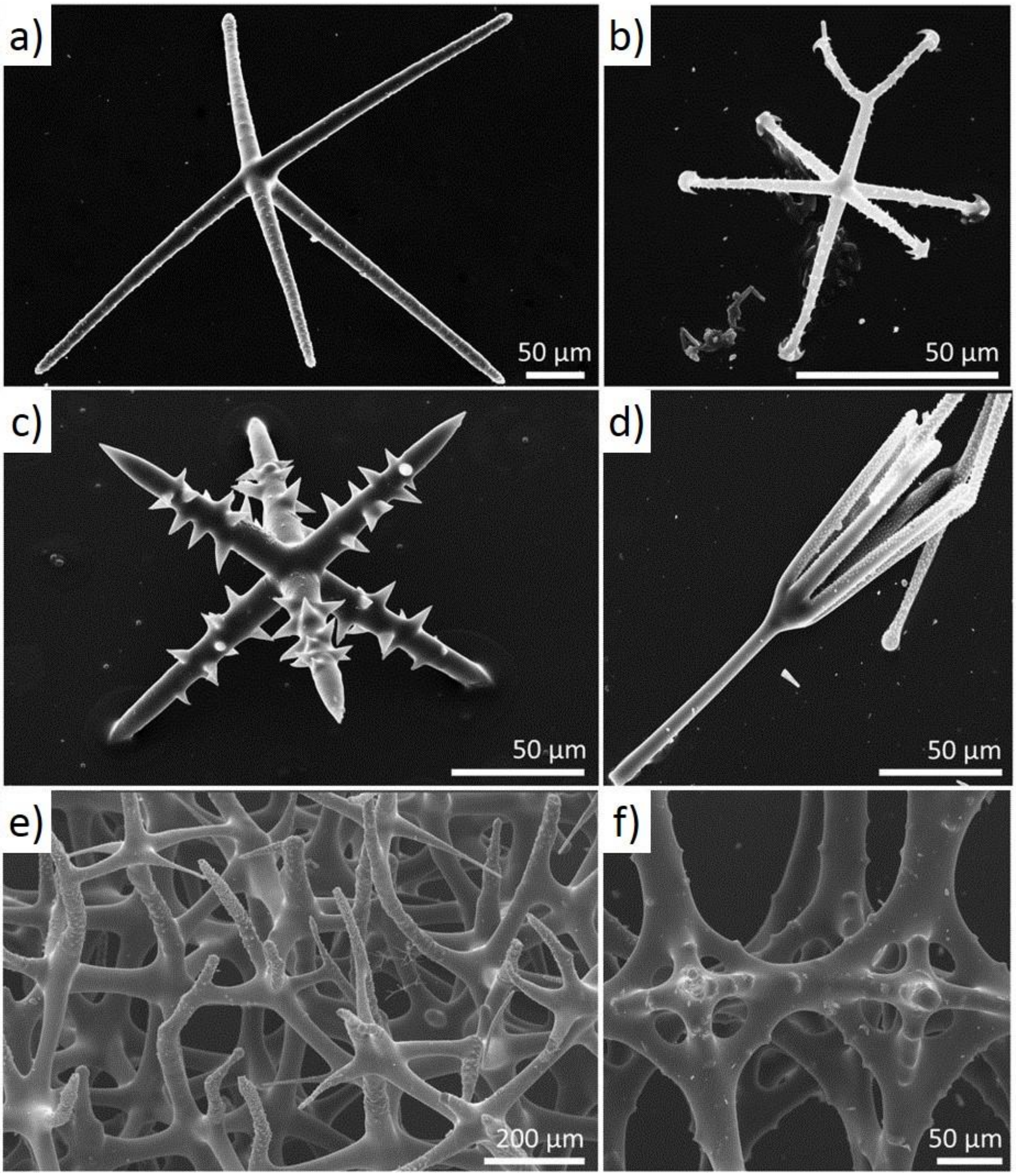

Figure S4. Structural diversity of selected skeletal structures in glass sponges (Hexactinellida). SEM images represent both individual structures (**a**, pentactin; **b**, hemidiscohexaster; **c**, spiny hexactin; **d**, scopule), as well as fused 3D constructs (**e**, euretoid dictyonal skeleton and **f**, lychniscosan dictyonal skeleton).

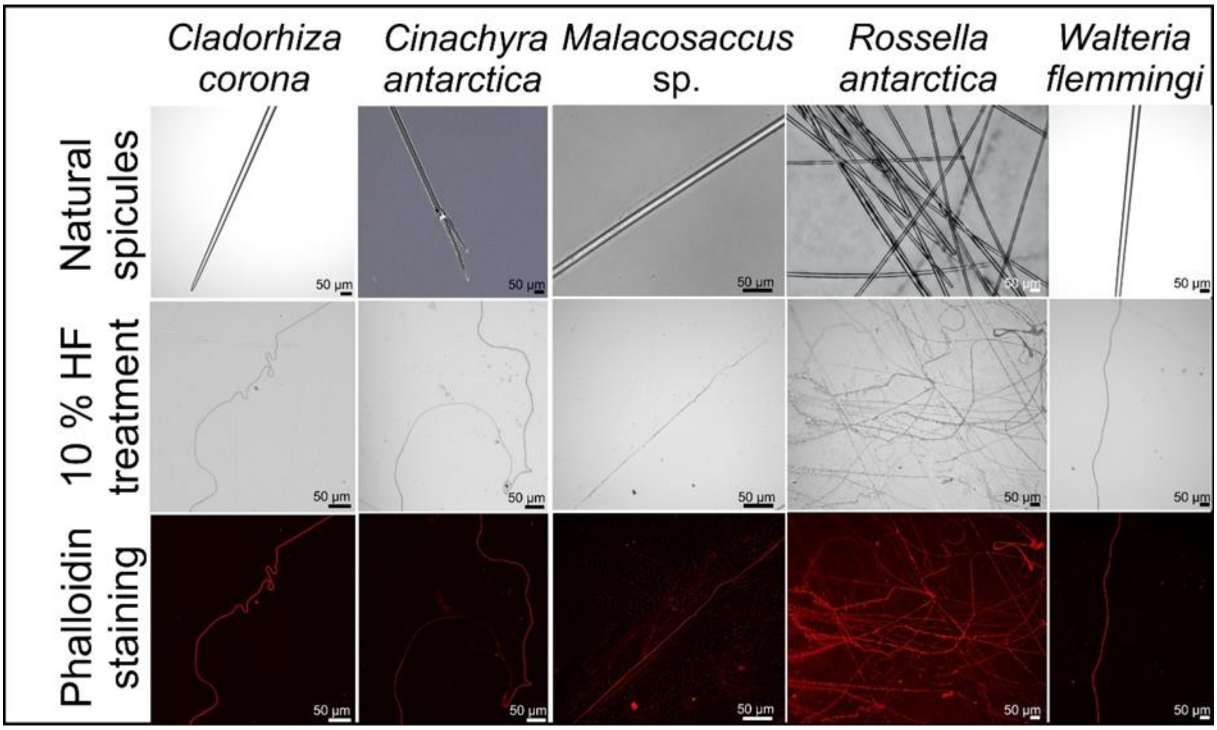

Figure S5. Overview of stained axial filaments**.** iFluor 594-Phalloidin stained axial filaments were isolated using HF-based treatment from spicules of selected demosponges (*Cladorhiza corona, Cinachyra antarctica*) and glass sponges (*Malacosaccus* sp., *Rossella antarctica* and *Walteria flemmingi*).

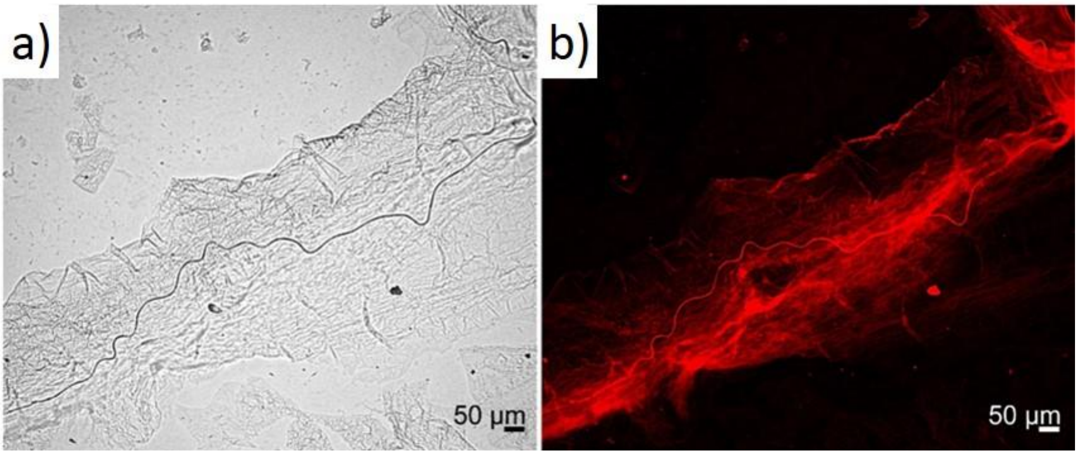

Figure S6. Visualization of axial filaments isolated from *Hyalonema (Corynonema) populiferum*. Due to it higher density in contrast to surrounding, diffuse organic matrix, the axial filament from *Hyalonema (Corynonema) populiferum* glass sponge anchoring spicules (isolated using 10% HF treatment at RT) becomes visible on the light microscopy image (**a**). The background of the organic matrix stained with iFluor 594-Phalloidin is indicative of actin presence (**b**).

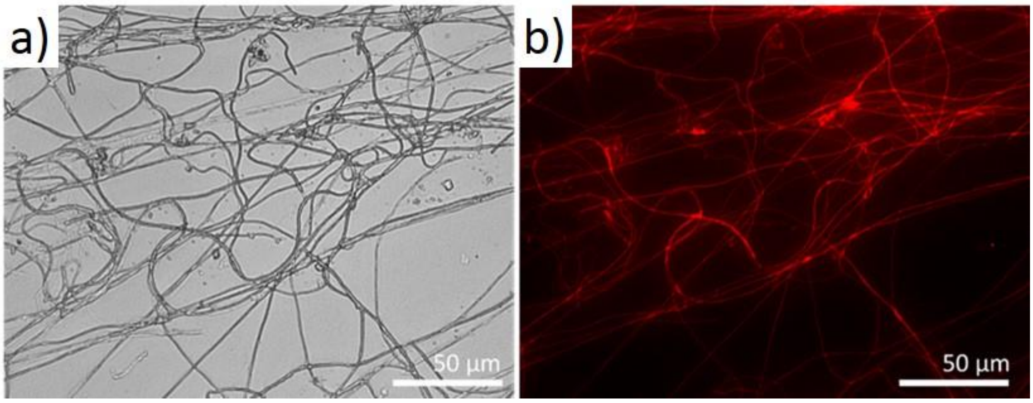

Figure S7. Visualization of axial filaments isolated from *Hyalonema sp.* Light (**a**) and fluorescence microscopy (**b**) images of the axial filaments isolated from 50 cm-long anchoring spicules of *Hyalonema* sp. glass sponge. Identification of actin within these axial filaments was carried out using iFluor 594-Phalloidin (**b**).

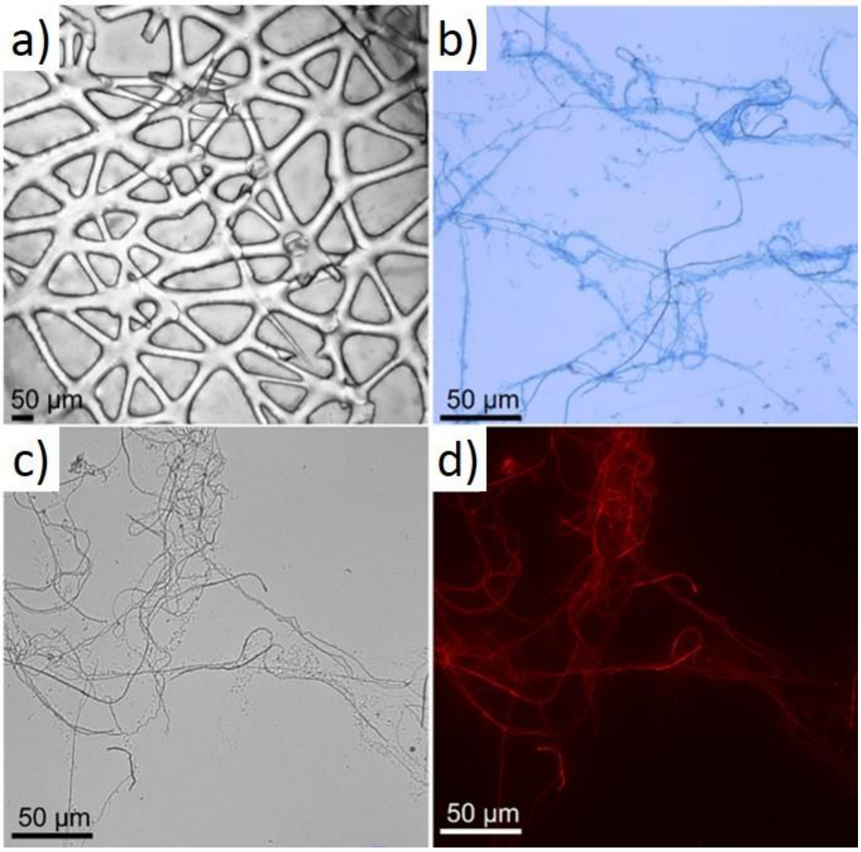

Figure S8. Visualization of axial filaments isolated from *Aphrocallistes beatrix***.** HF treatment of the fragments of siliceous skeletal network isolated from *Aphrocallistes beatrix* glass sponge (**a**), yields proteinaceous axial filaments, stained with Coomassie blue G-250 (**b**). These axial filaments (**c**) also show intensive red fluorescence (**d**) after staining with iFluor 594-Phalloidin, used for actin identification.

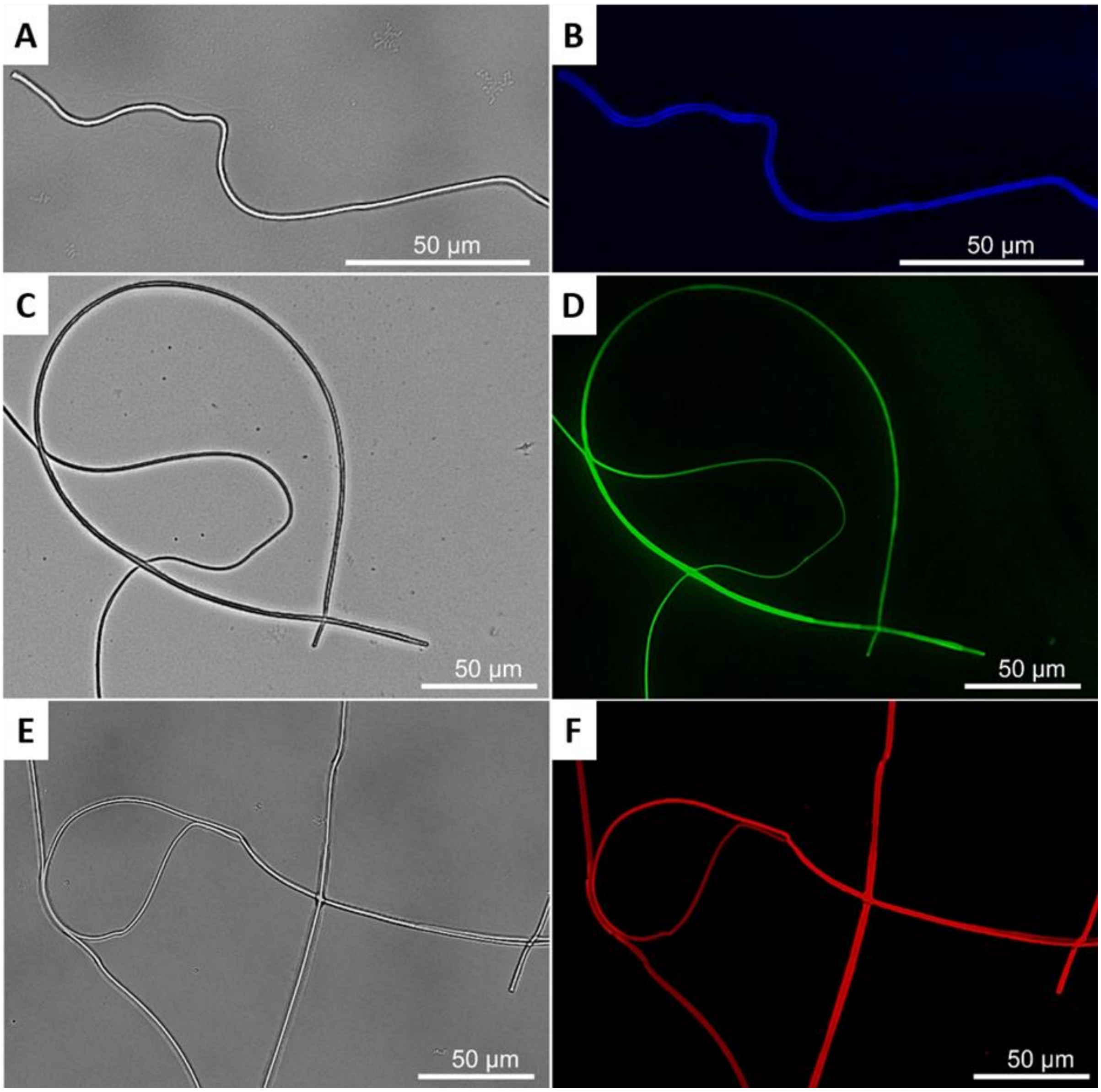

FigureS9. Visualization of axial filaments isolated from oxeas of *Geodia cydonium*. Light (**a, c, e**) and fluorescence microscopy (**b**, **d**, **f**) images of the axial filaments isolated from oxeas of *G. cydonium* and stained with iFluor™ 350-Phalloidin (blue fluorescence), iFluor™ 488-Phalloidin (green fluorescence) and iFluor™ 594-Phalloidin (red fluorescence) as indicators of actin.

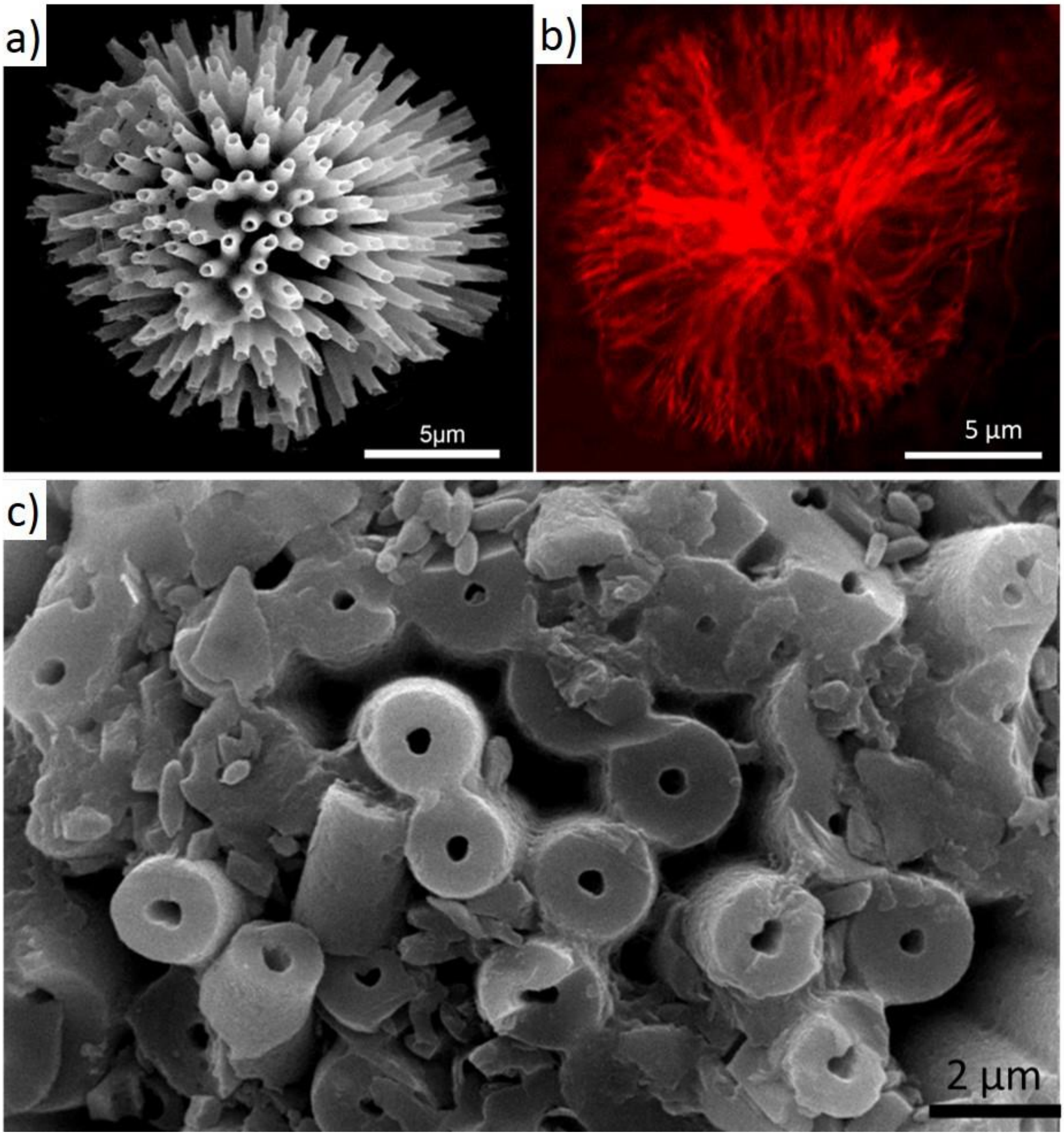

Figure S10. Visualization of axial filaments isolated from *Geodia cydonium* oxyasters. HF-based treatment of siliceous spherical microspined rays constructing oxyasters of *G. cydonium* marine demosponge (SEM image, **a**) led to the isolation of unique organic matrix in the form of radial actin bundles, which also became visible following iFluor 594-Phalloidin staining (fluorescence microscopy image, **b**). Such radial actin pattern upon radially symmetric growth of up to 8 µm long actin bundles remains unique. Occurrence of morphologically similar F-actin aster-like structures has been recently reported in early neuronal development [57]. SEM image (**c**) of the mechanically disrupted oxyasters shows broken siliceous rays with corresponding axial channels, in which axial filaments have been initially located.

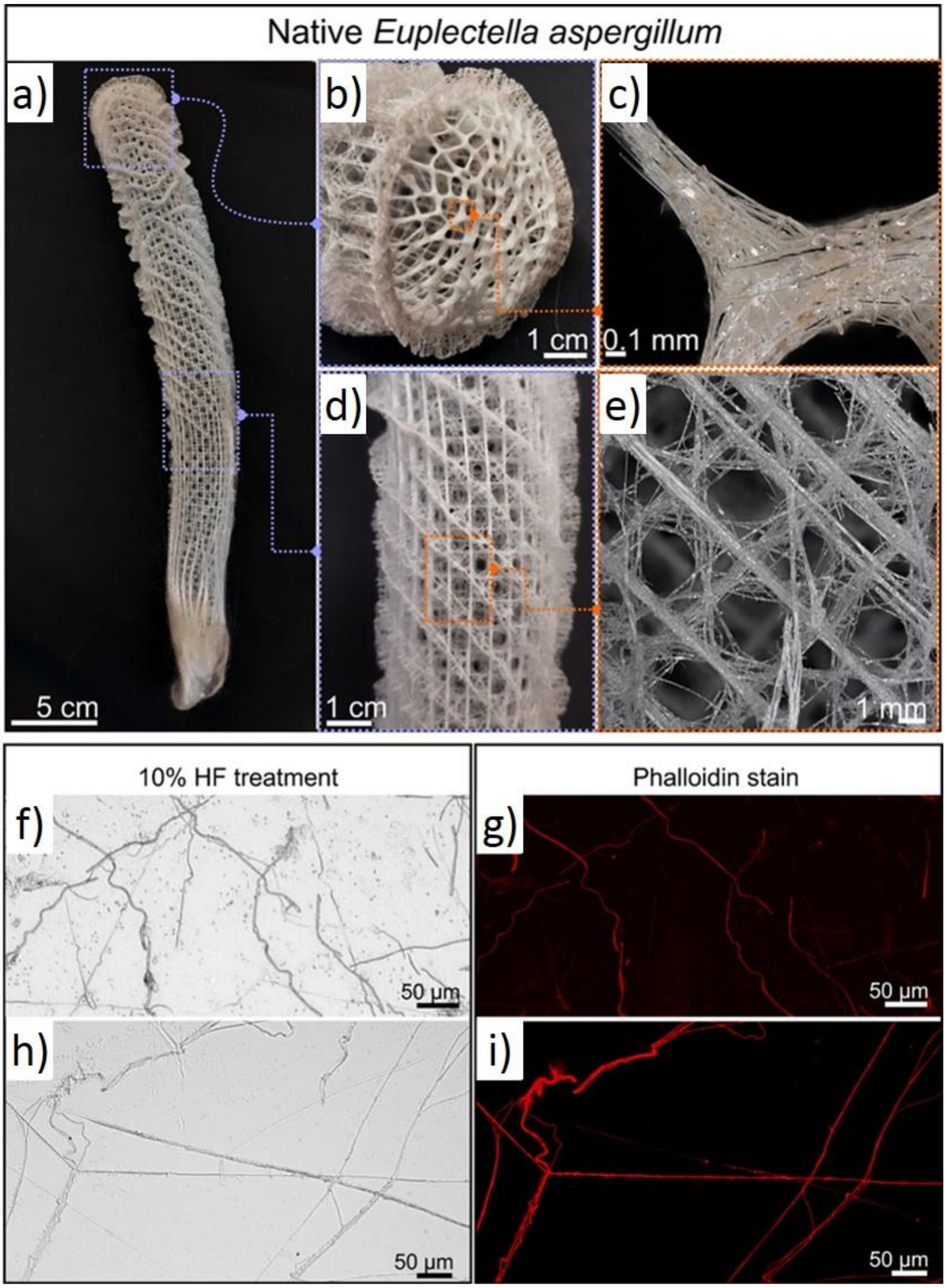

FigureS11. Visualization of axial filaments isolated from *Euplectella aspergillum*. Hierarchical and complex skeletal architecture *of E. aspergillum* glass sponge (**a, b, c, d, e**) remains to be the classical research object for materials scientists. HF treatment leads to isolation of axial filaments (light microscopy images **f** and **h**) from the skeletal framework after 72 h. In spite of fragility, some of axial filaments still resemble the structural motives with geometries (**h**) known for intact skeleton (**e**). Specific iFluor™ 594-Phalloidin staining identifies their actin nature (**g**, **i**).

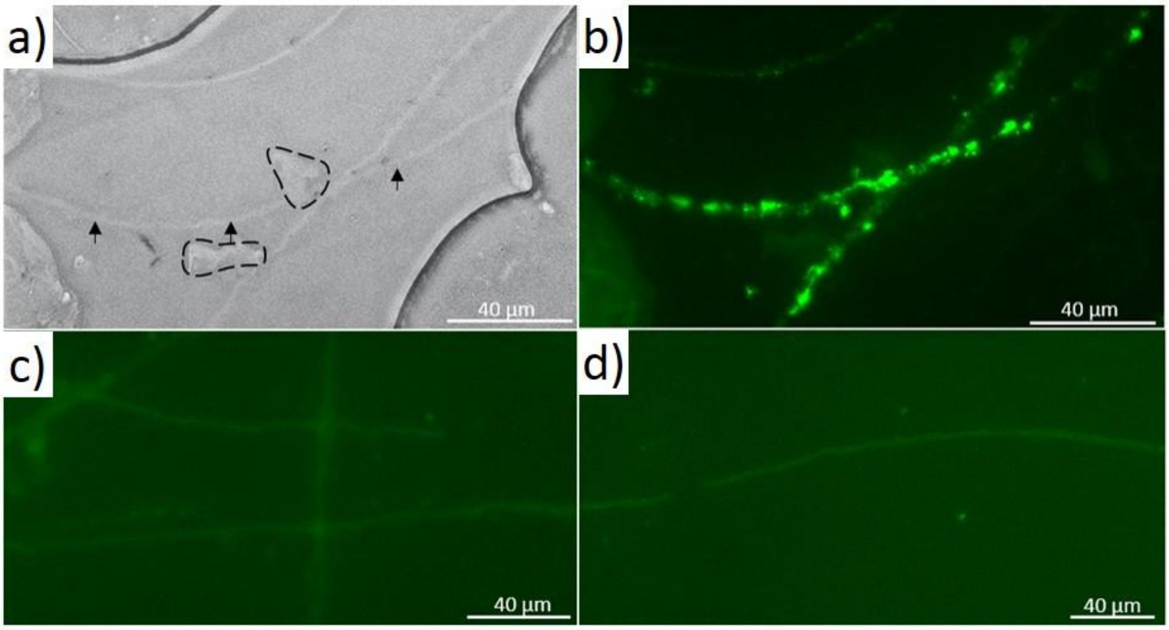

Figure S12. Identification of actin within axial filaments of glass sponge skeleton using immunostaining. Light microscopy image (**a**) of the partially demineralized skeletal framework of *Euplectella* sp. glass sponge after immunostaining. Dotted lines represent the micro fragments of residual silica, and the arrows show the axial filaments. The results of immunostaining of the same sample with respect to actin is represented in the image (**b**). Treatment of such axial filaments only with secondary antibodies (**c, d**) show only weak autofluorescence in comparison to image (**b**).

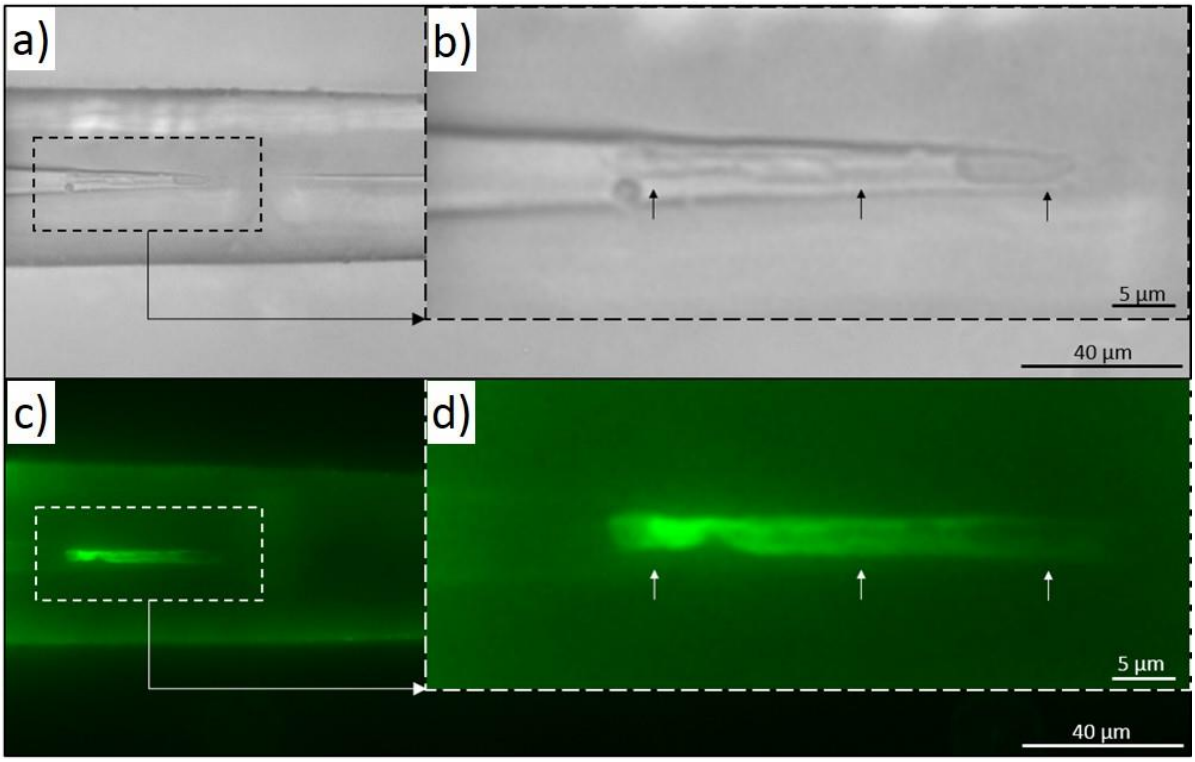

Figure S13. Identification of actin within axial filaments within demosponges spicules using immunostaining. Light microscopy images (**a, b**) of partially demineralized and immunostained with respect to actin identification oxeas of *G. cydonium* marine demosponge show well- preserved axial filament. Fluorescence microscopy images (**c, d**) of this sample strongly confirm the presence of actin.

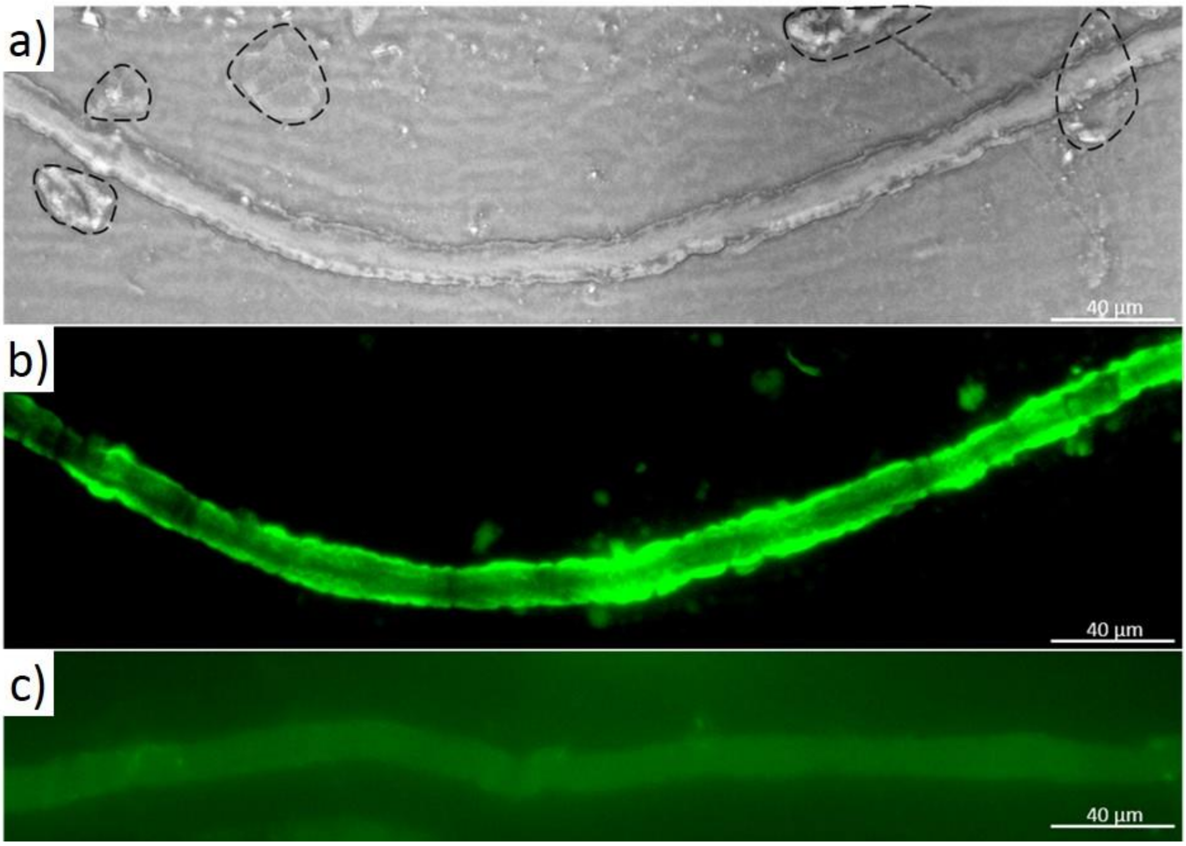

Figure S14. Identification of actin within axial filaments of glass sponges spicules using immunostaining. Light microscopy image (**a**) of the partially demineralized spicule of *Hyalonema* sp. glass sponge after immunostaining. Dotted lines represent the micro fragments of residual biosilica. The results of immunostaining of the same sample with respect to actin is represented in the image (**b**). Treatment of such axial filaments only with secondary antibodies (**c**) show only weak autofluorescence in comparison to image (**b**).

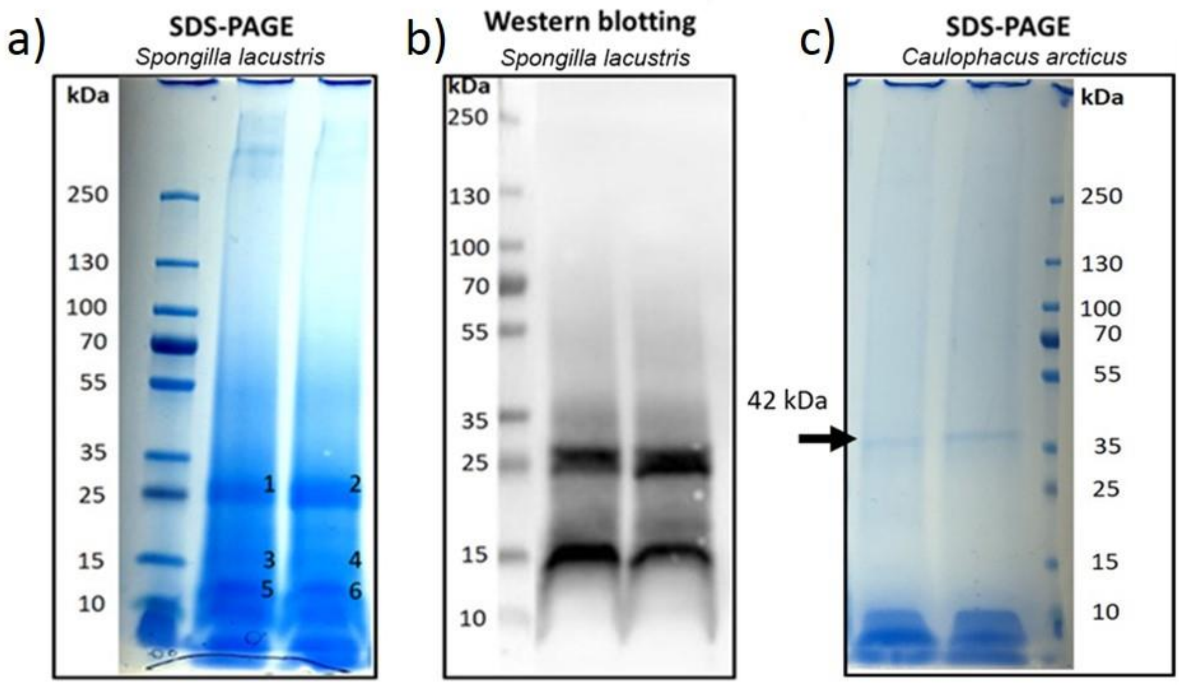

Figure S15. Identification of actin in the axial filament of *Spongilla lacustris* and *Caulophacus arcticus* sponges. The proteins isolated from the axial filament of *S. lacustris* and *C. arcticus* sponges were separated using the SDS-PAGE and either visualized by Coomassie G-250 staining (**a**, **c**) or transferred to the PVDF membrane and traced using β-actin specific antibodies (**b**). (**a**) The corresponding bands from *S. lacustris* SDS-PAGE were cut out, trypsin digested and identified by mass spectrometry as actin (bands 1 and 2) and silicatein (bands 1-6). (**c**) SDS-PAGE from *C. arcticus* indicated the presence of dominating 42 kDa band. Results derived from LC-MS/MS analysis are presented in **Tables 4** and **5,** Supporting Information.

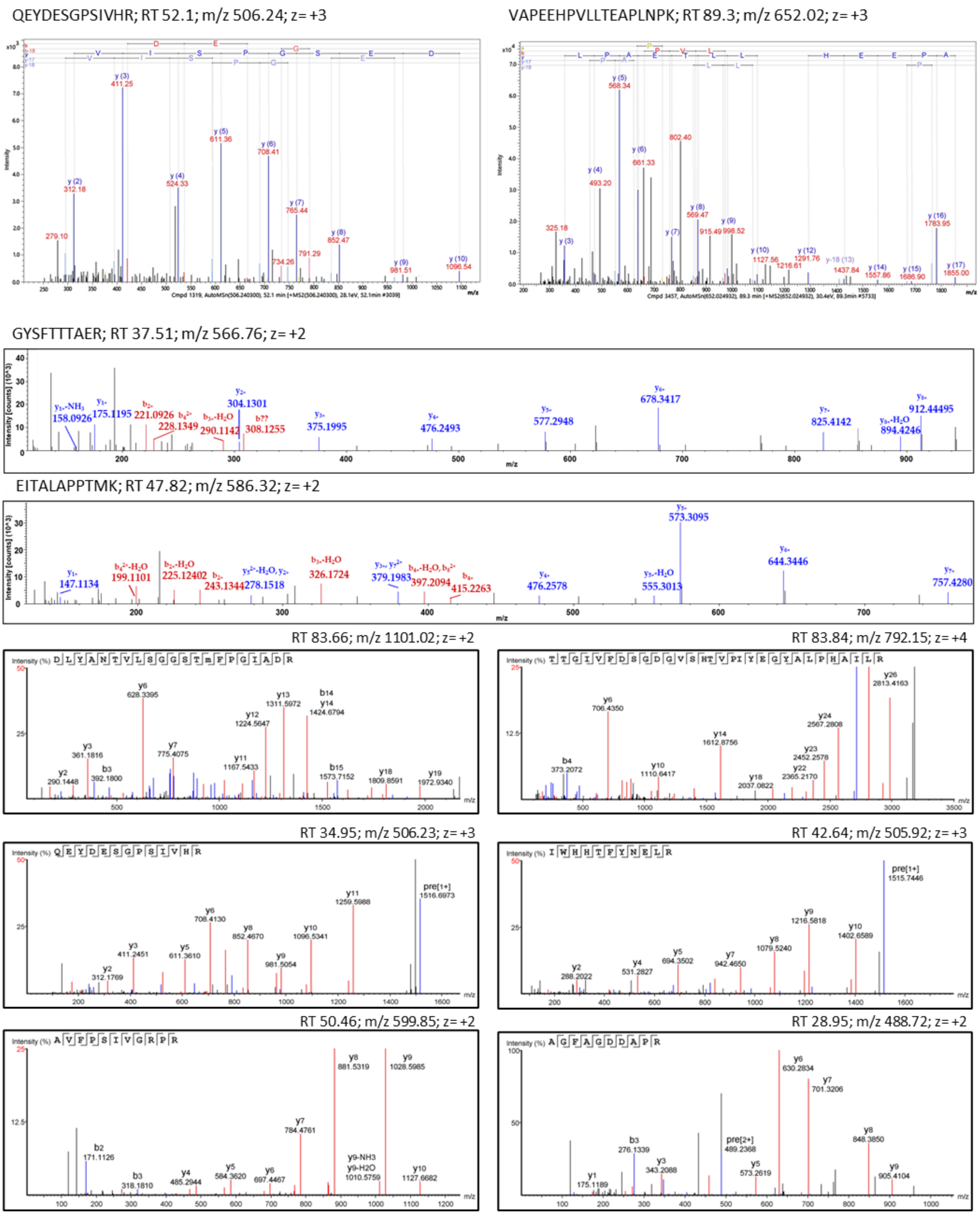

Figure S16. LC-MS/MS analysis of actin. Representative MS/MS spectra of tryptic peptides identified as actin in axial filaments of *C. arcticus*, *S. lacustris*, *M. chuni* and *M. sp*. sponges. Retention time (RT), precursor mass (*m/z*), charge (z) and carbamidomethyl / oxidation modifications are presented.

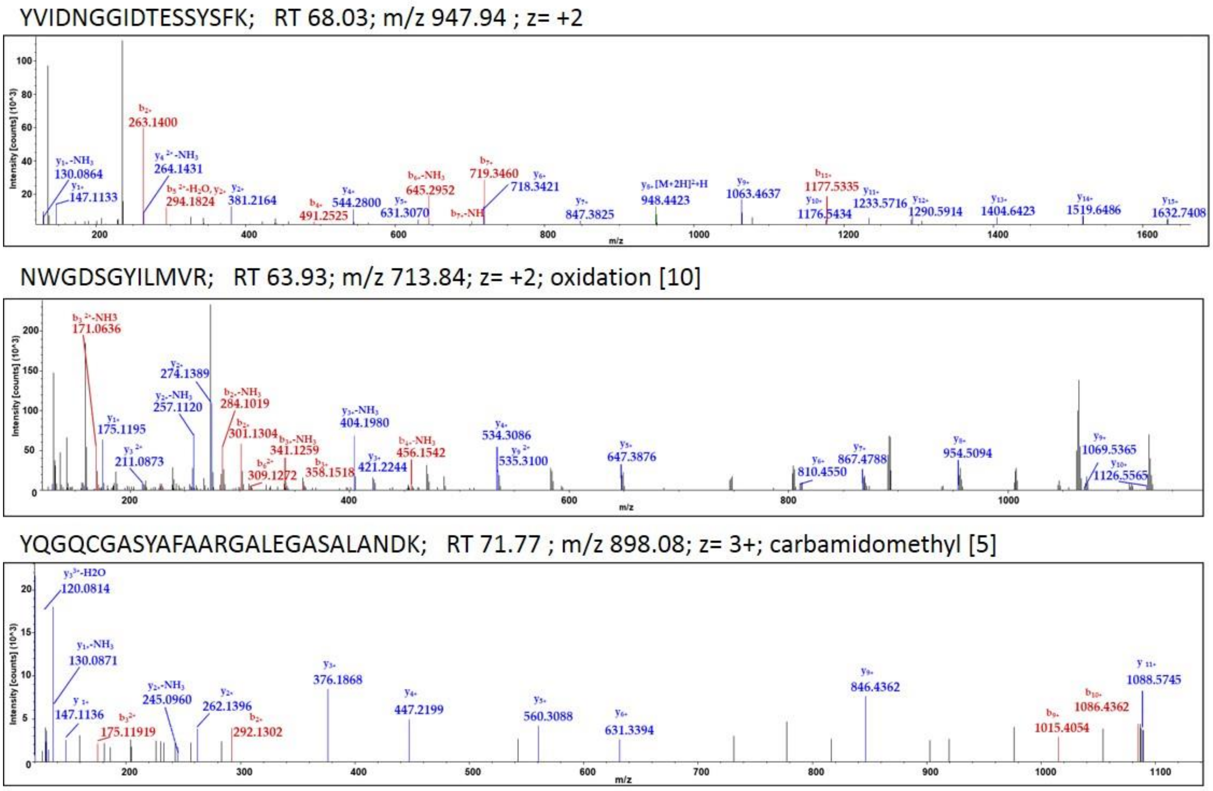

Figure S17. LC-MS/MS analysis of silicatein. Representative MS/MS spectra of tryptic peptides identified as silicatein in axial filaments of *S. lacustris* freshwater demosponge (see **Table 5**, Supporting Information). Retention time (RT), precursor mass (*m/z*), charge (z) and carbamidomethyl / oxidation modifications are presented.

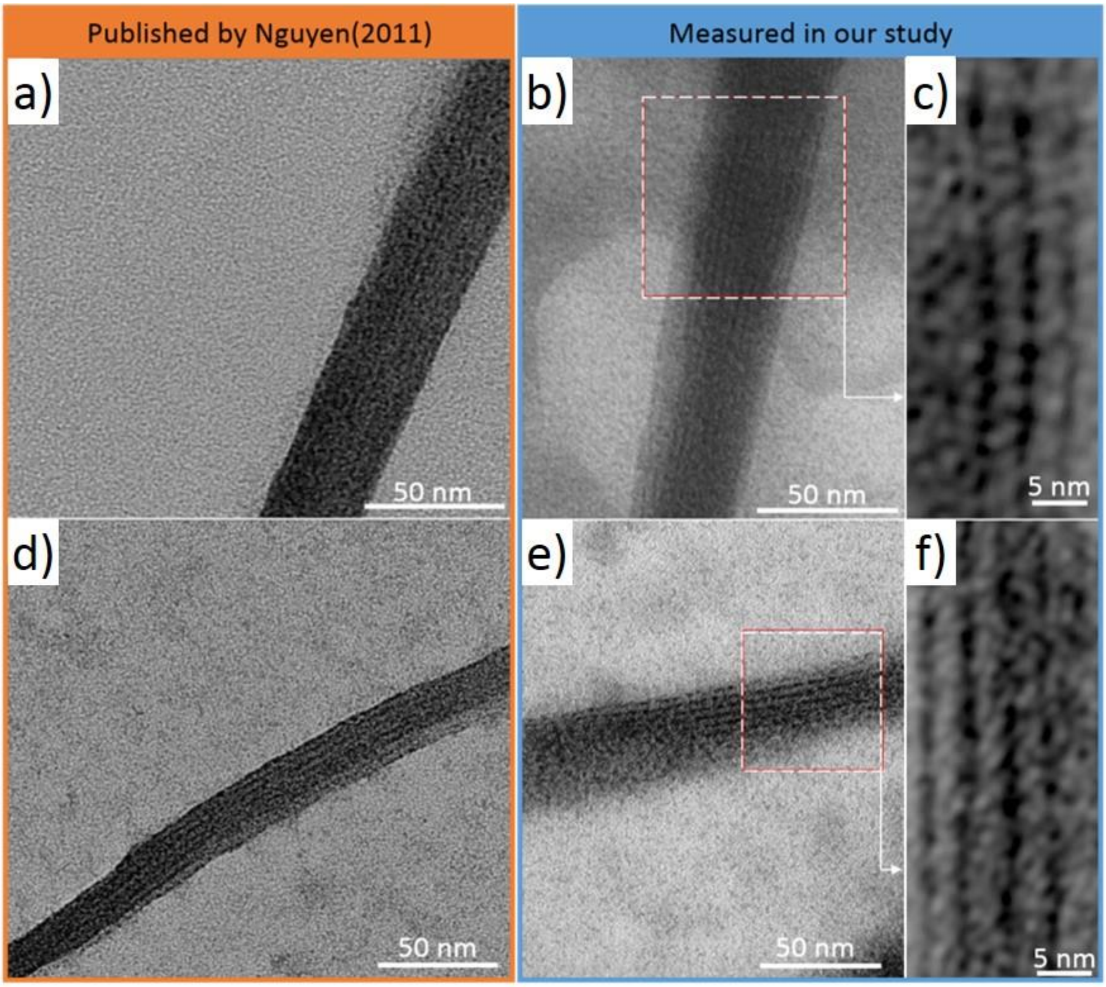

Figure S18. Structural similarity between F-actin bundles and nanofibers of axial filament of *Monorhapis chuni* glass sponge**.** TEM images (**a**, **d**) of the bundles of F-actin standard observed previously [54] show high similarity to that obtained by us for selected nanofibers of *M. chuni* axial filament (**b**, **e**). The magnification shows double helices typical for actin filaments (**c**, **f**).

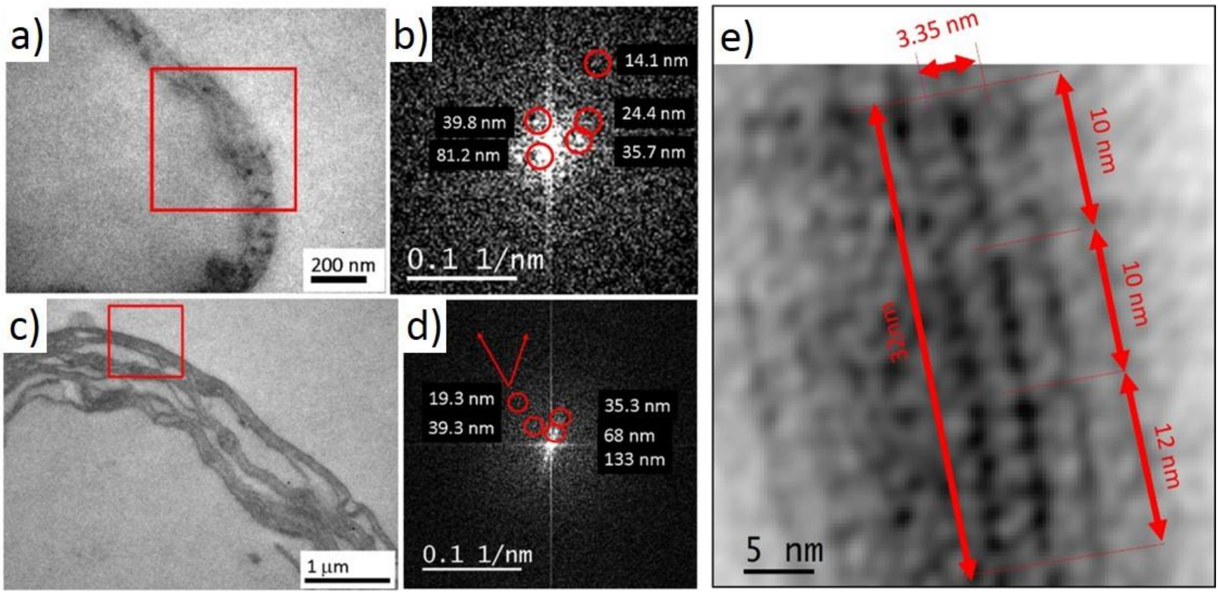

Figure S19. TEM and Fourier analyses of the axial filaments isolated from glass sponges. Fragments of axial filaments of glass sponges *M. chuni* (**a**) and *C. arcticus* (**c**) where the fibril length amounts to 1.2 µm and the width varies between 90 nm to 140 nm. The FFT in **Figure S19b**, Supporting Information taken from the red frame area of **Figure S19a** indicates different large periodicities typical for actin such as 39.8 nm and 35.7 nm, see [59-61]. This value is due to a 28/13 symmetry, or a helical repeat of 28 subunits in 13 turns of the 59 Å. Spacing of 81.2 nm is close to the value that Poole et al. [58] registered at 77 nm. We propose this spacing corresponds to the first order reflection of the actin spacings around 39 nm according to 77nm/2 giving 38.5nm. The typical reflection of 14 nm for actin was also detected, (see *e.g.*[63,64]), corresponding to the distance between the centres of neighbouring actin filaments. **Figure S19c** shows a bundle of actin filaments about 800 nm thick, subdivided to 140 nm thin sub-fibrils. The FFT of the fibrils of the red marked area of (**c**) show reflections corresponding to spacings of 19.3 nm, and 39.3 nm in one direction corresponding to a first and second order reflection. In the other direction we observed spacings of 133 nm, 68 nm and 35.3 nm, which represent the first through third order reflections of actin. The two directions are visualized by arrows. **e**) HRTEM analysis of the surface of an individual fibril of *M. chuni* axial filament (see also **Figures S18** and **S22**, Supporting Information) shows structural features typical for mono-filament-bound cross-links observed previously from actins of diverse origin *[65]*.

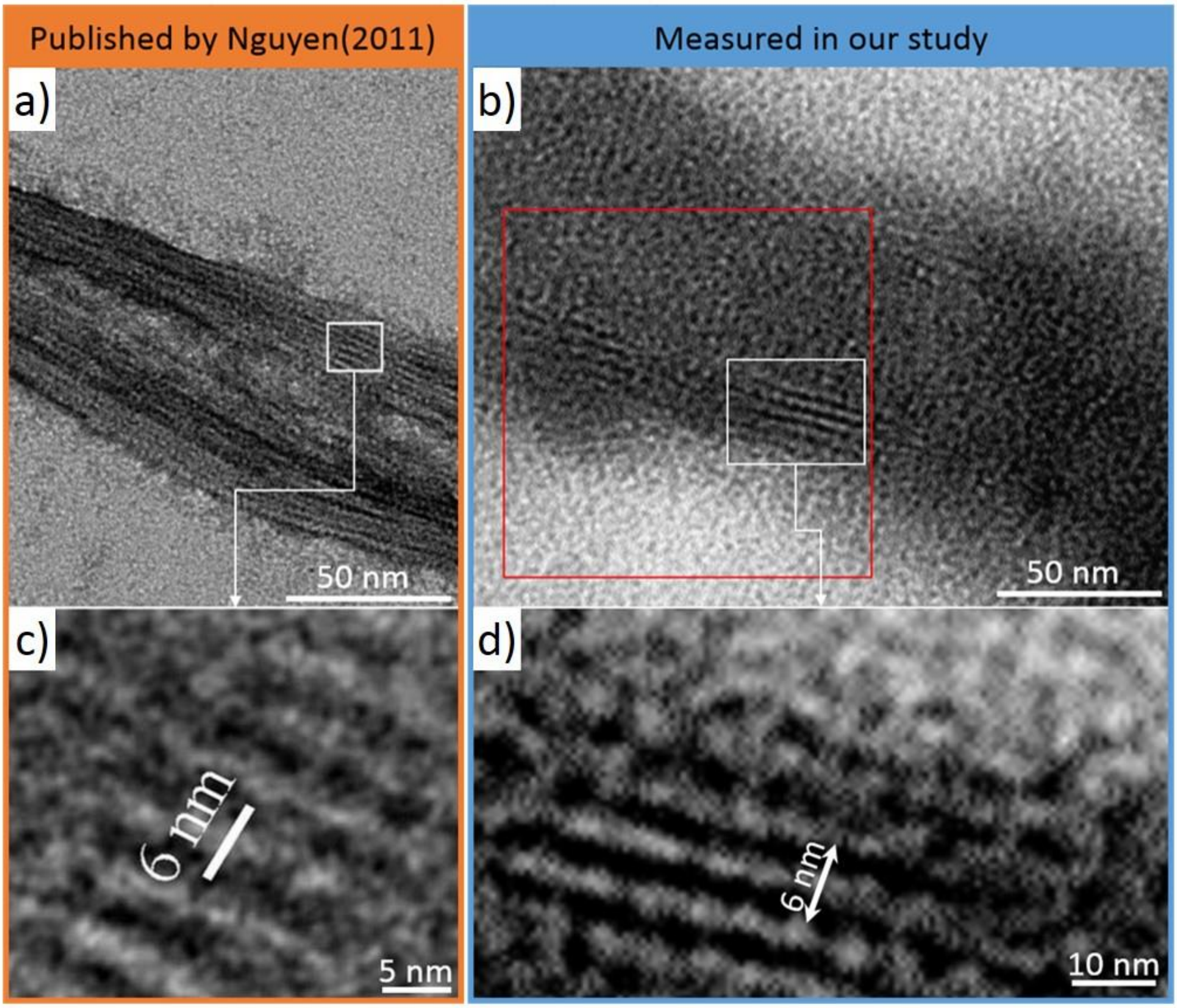

Figure S20. Structural similarity between F-actin bundles and nanofibers of *Monorhapis chuni* axial filament. TEM images (**a**, **c**) of the bundles of F-actin standard observed previously [58] show high similarity as compared to that obtained by us for selected nanofibers of *M. chuni* axial filament (**b**, **d**).

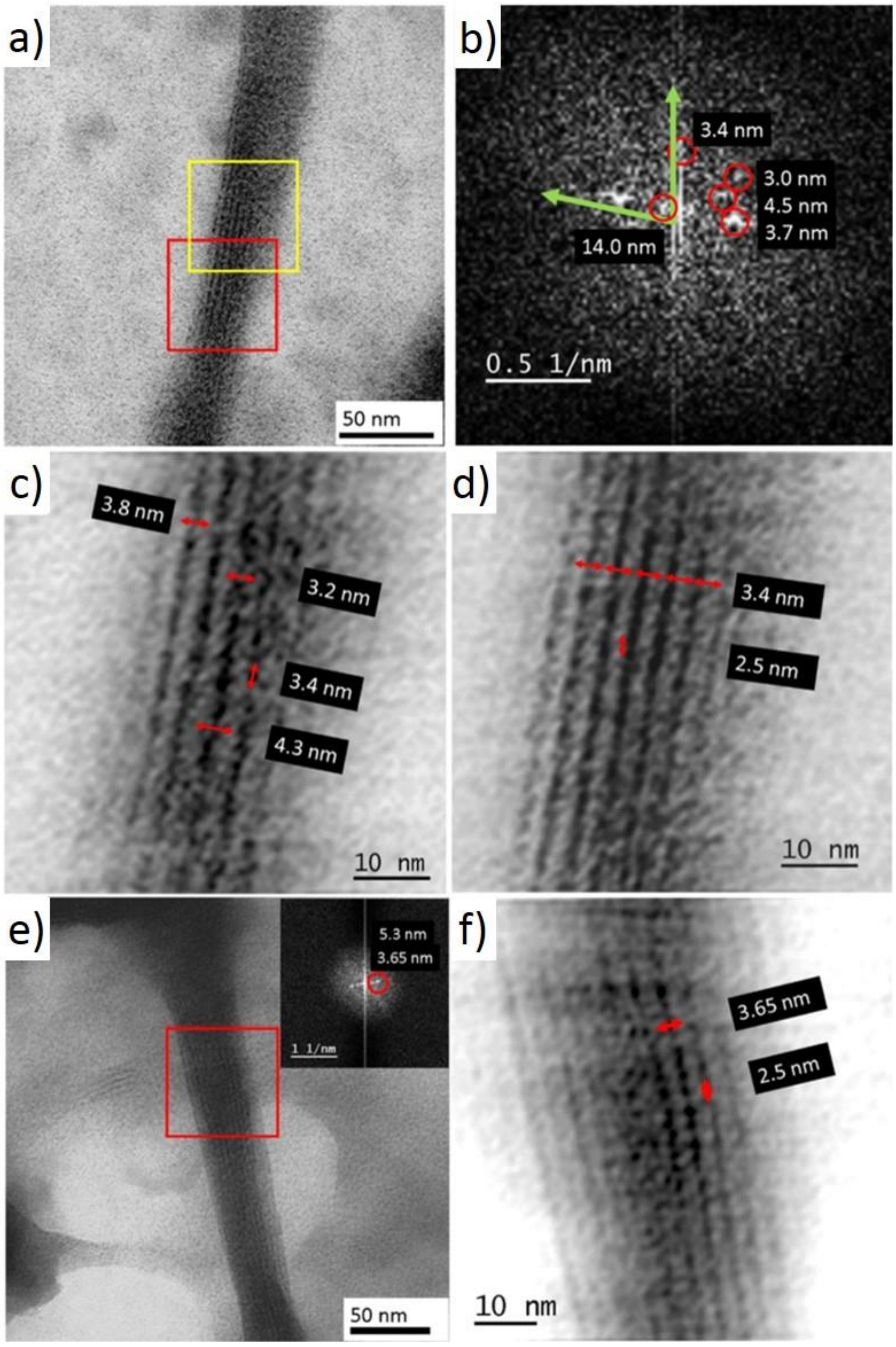

Figure S21. Bridging actin filaments of *Monorhapis chuni* axial filament. (**a**) Individual axial filament fibril of 25 nm diameter (centre part). (**b**) FFT shows typical spacings between 3.0–4.5 nm from red rectangle ROI shown in (**a**). (**c**) Noisy background filtered image of central part indicated as red frame in (**a**). (**d**) Upper central part (yellow frame in (**a**)), background filtered showing a more regular structuring with 3.4 nm spacing between fibrils and 2.5 nm periodicity along the fibril. (**e**) Bridging actin filament with FFT inset at right top indicating periods of 3.65 nm and 5.3 nm parallel to fibril long axis. (**f**) Magnified and filtered image of

red marked area in (**e**). Typical for actin spacings between single fibrils of 3.65 nm and 2.5 nm periodicity along the fibril are well visible (see **Table 7,** Supporting Information).

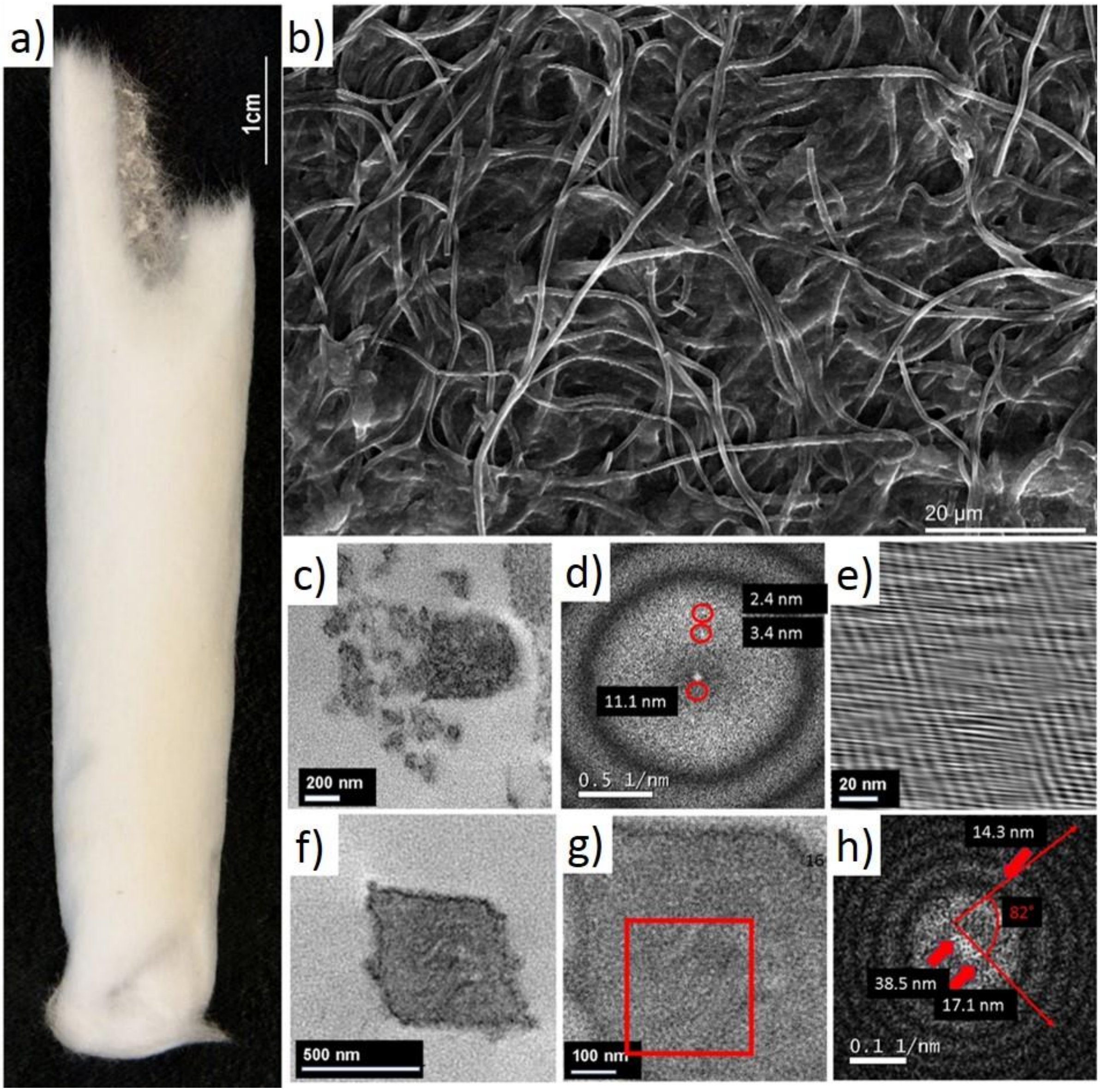

Figure S22. FFT analytics. Overview (**a**) of the anchoring stalk of *Caulophacus arcticus* glass sponge from which axial filaments represented on SEM image (**b**) have been isolated. (**c**) TEM overview of non-stained ultrathin cross-section of selected fragment of the *C. arcticus* actin filament. (**d**) FFT reveals spacings of 11.1 nm, 3.4 nm and 2.4 nm. 11.1 nm is fitting to the *c*-axis value of (001) [66] whereas 3.4 nm corresponds either to (003) or (103) and 2.4 nm to (201) or (202) of actin filaments see [63, 67]. As reported previously [68], the spacing at 2.4 nm is strongly related to the actin helix. (**e**) Fourier filtered image of a selected magnified region of interest of (**c**) confirms this size for individual actin helices very well. (**f**) Overview of another non-stained ultrathin cross section of *C. arcticus* axial filament with diameter of about 600 x 600 nm. (**g**) Zoom into (**f**) with region marked in red for fast Fourier transform (FFT) analysis. (**h**) FFT of (**g**) showing spacings of 38.5m, 17.1 and 14.3 nm. Such spacings as 38.5 nm and 14.3 nm have been reported previously as typical for actin [60, 61, 64, 67], respectively) (see **Table 7,** Supporting Information).

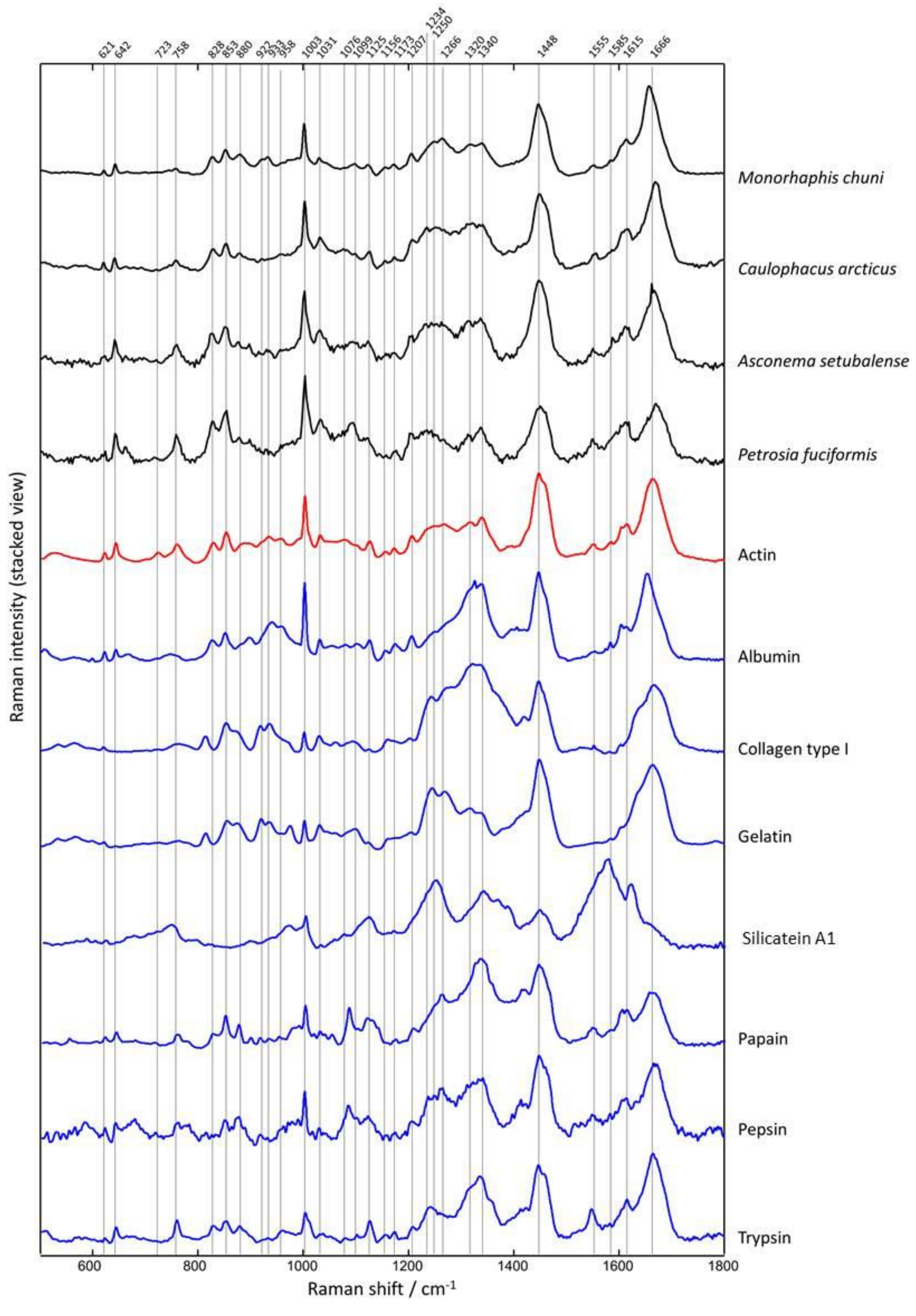

Figure S23. Comparative Raman spectroscopy of isolated axial filaments. Raman spectra of the standard reference (actin from rabbit muscle, Sigma Aldrich A2522) (red line) in comparison to that obtained for selected axial filaments extracted from glass sponges *Monorhaphis chuni*, *Caulophacus arcticus*, *Asconema setubalense* and from demosponge *Petrosia fuciformis* (black lines) as well as standards of non-actin related proteins such as albumin, collagen I, gelatin, silicatein A1, papain, pepsin and trypsin (blue lines). Position and assignment of Raman bands of actin is represented in the **Table 8,** Supporting Information.

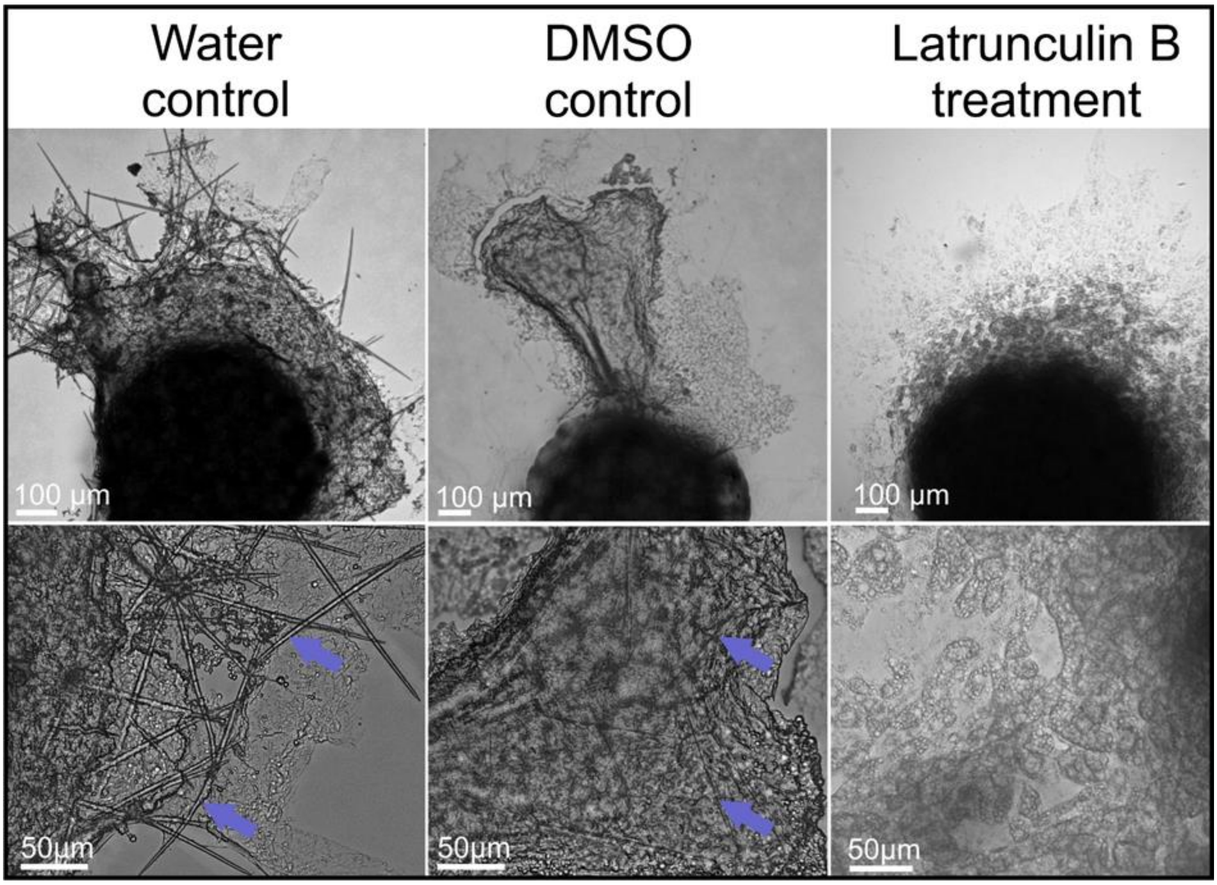

Figure S24. Inhibition of spiculogenesis in model experiment. *S. lacustris* freshwater demosponge has been employed for the model experiment due to its ability to produce dormant gemmules which, upon hatching, individually produce a new small sponge. Light microscopy images represent the spicules (arrows), which appear during the hatching of *S. lacustris* gemmules and initial stage of the young sponge formation in the presence of water and DMSO. No spicules were observed in experiments under same conditions where latrunculin B as inhibitor of actin polymerization has been added. See also **Figure 5** in the main text.

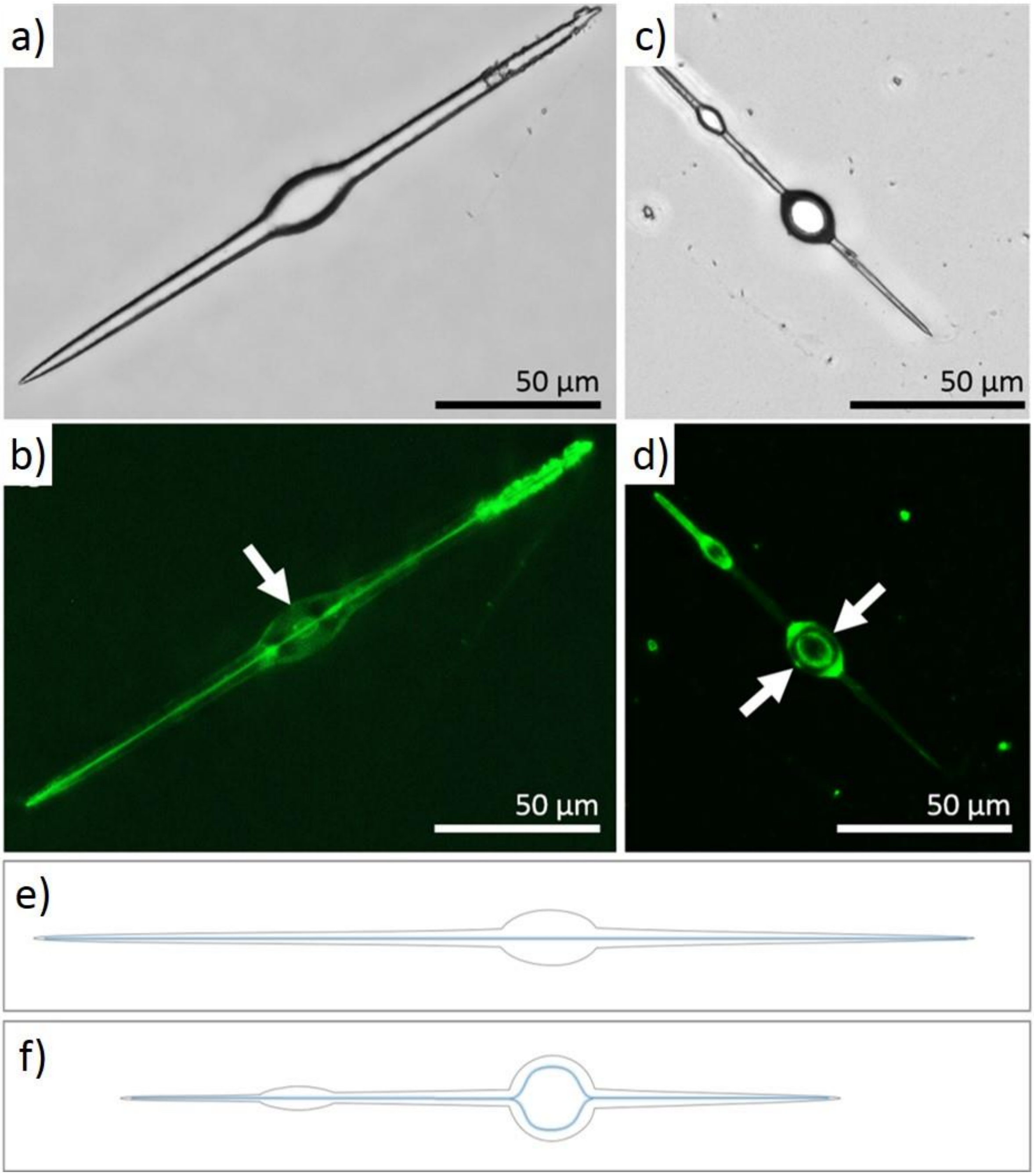

Figure S25. Structural peculiarities of actin filaments in *Spongilla lacustris* demosponge spicules under influence of germanium. Addition of GeO_2 into growth medium of *S. lacustris* demosponge under 0.5 Ge/Si-ratio leads to drastically changes in spicule's morphology with respect to appearance of bulbs **(c**, **d**) and reduction of spicule length in comparison to Ge-free control (**a**, **b**). The fluorescence microscopy images (**b**, **d**) strongly confirm that formation of the malformed spicules (**c**) is attributed to the bifurcation (**d**, arrows) of the actin-based (**Figure 1**) axial filament as comparatively represented on the schematic presentation of actin filaments trajectories (**e**, **f**). In contrast to other fibrillar proteins (i.e. collagen), structural branching is attributed to the one of characteristic features of the actin filament growth in diverse organisms (see for review [69]).

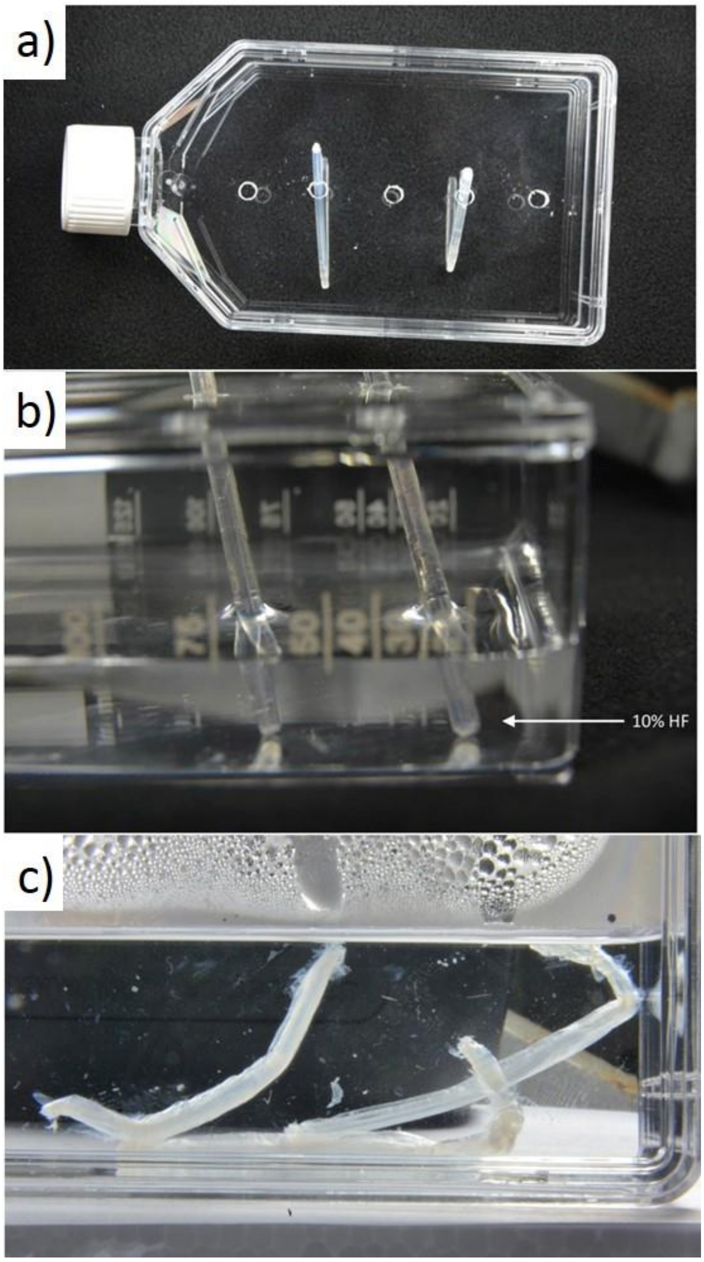

Figure S26. Isolation of axial filaments from anchoring spicules of *Monorhaphis* sp. (**a**) in 10% HF at RT in plastic vessel (**b**, **c**) remains to be simple procedure, which leads to appearance of centimetre-long and up to 3 mm thick silica-free organic constructs (**c**) after 72 h of demineralization.

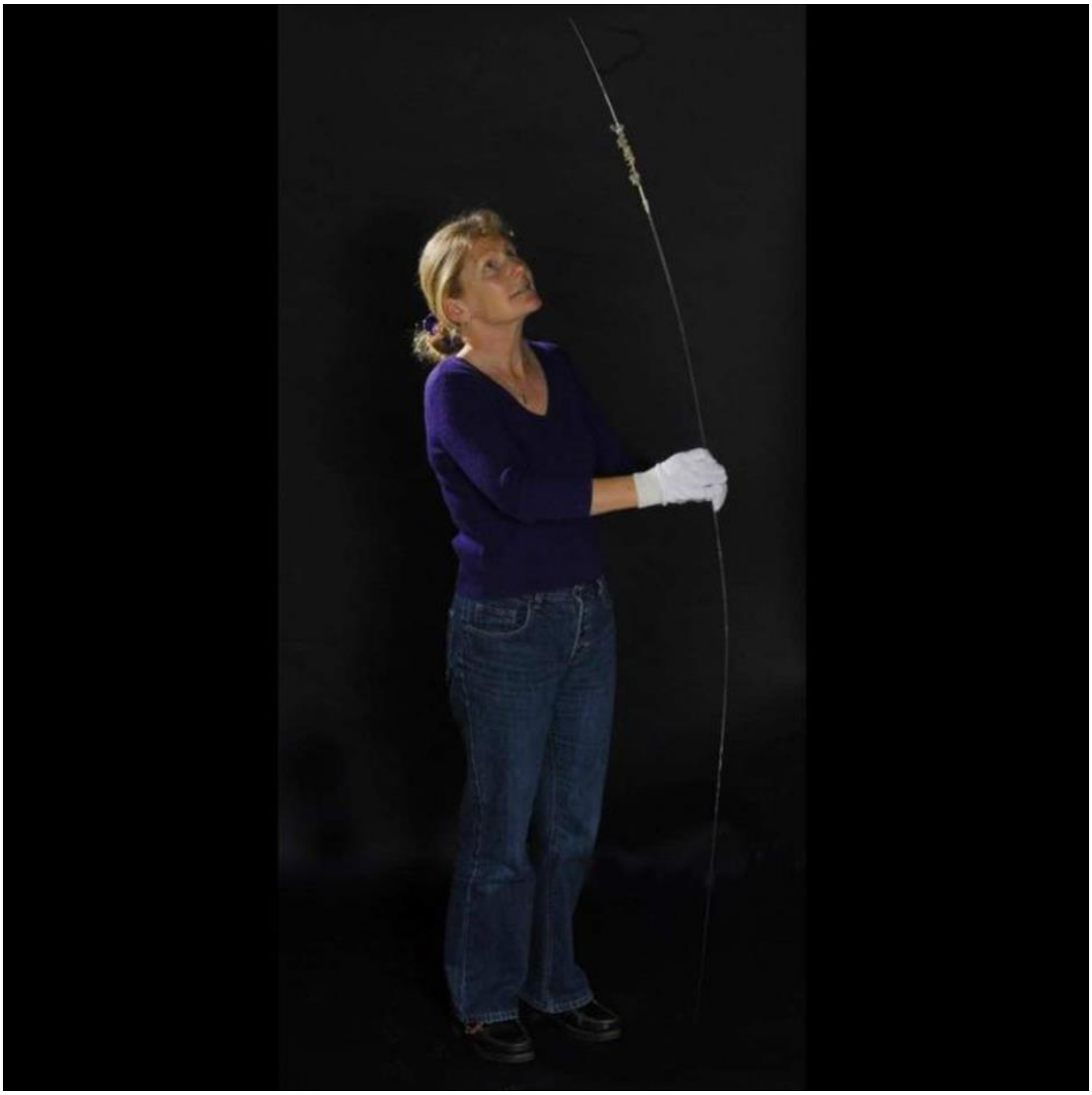

Figure S27. Giant anchoring spicule**.** Glass sponge *M. chuni* is able to produce the longest biosilica structure ever reported in the form of individual anchoring spicule with length up to 3 meter. Image courtesy Western Australian Museum (Perth, Australia).

Table 1. Identification of actin in the axial filament of *Asconema setubalense* glass sponge. The proteins extracted from the axial filament of *A. setubalense* were separated using the SDS-PAGE and trypsin digested (**Figure 3c**) or directly digested and analyzed by label-free nanoLC-MS/MS approach. Seven actin (A0A1Y9T597) peptides were identified with 27.13% sequence coverage after label-free approach. Four of them (marked with gray sections) were identified after SDS-PAGE separation. The same peptides were identified as derived from actin in other species: *Strongylocentrotus franciscanus* (P10990), *Limulus polyphemus* (P41340) or *Dictyostelium discoideum* (Q554S6).

Actin peptide sequence	Mass	m/z	Δ [ppm]	z	RT	Modification
**VAPEEHPVLLTEAPLNPK**	1953.07	652.02	3.68	3	57.93	
**TTGIVFDSGDGVSHTVPIYEGYALPHAILR**	3184.64	797.16	6.79	4	87.45	
**AVFPSIVGRPR**	1197.70	400.23	2.32	3	57.81	
**EITALAPPTMK**	1170.63	586.31	0.01	2	51.27	
**GYSFTTTAER**	1131.53	566.76	2.75	2	38.53	
**DSYVGDEAQSK**	1197.52	599.76	3.65	2	22.80	
**DSYVGDEAQSKR**	1353.62	677.81	3.38	2	21.89	

Table 2. Identification of actin in the axial filament of *Monorhaphis chuni* glass sponge. The proteins extracted from the axial filament of *M. chuni* were trypsin in-solution digested and analyzed by label-free nanoLC-MS/MS approach. Fourteen actin (A0A1Y9T597) peptides were identified with 45.7% sequence coverage.

Actin peptide sequence	Mass	m/z	Δ [ppm]	z	RT	Modification
**AGFAGDDAPR**	975.44	488.73	-0.2	2	29.11	
**DSYVGDEAQSK**	1197.51	599.76	0	2	25.81	
**DSYVGDEAQSKR**	1353.62	452.21	0.9	3	22.09	
**IWHHTFYNELR**	1514.74	505.92	0.4	3	43.56	
**MTQIMFETFNTPAMYVAIQAVLSLYASGR**	3300.59	1101.2	0.6	3	119.1	Oxidation (1,5)
**QEYDESGPSIVHR**	1515.70	506.24	-1.3	3	35.43	
**QEYDESGPSIVHRK**	1643.79	548.94	-3	3	26.87	
**SYELPDGQVITIGNER**	1789.88	895.94	-5.2	2	73.63	
**AVFPSIVGRPR**	1197.70	400.24	-0.7	3	50.47	
**DLTDYLMK**	1013.47	507.74	-2.6	2	62.5	Oxidation (7)
**GYSFTTTAER**	1131.52	566.77	-1.2	2	40.57	
**HQGVMVGMGQKDSYVGDEAQSK**	2384.03	597.01	-0.7	4	30.22	Deamidation (2,10) Oxidation (5,8)
**TTGIVFDSGDGVSHTVPIYEGYALPHAILR**	3184.62	797.16	-1.8	4	84.14	
**VAPEEHPVLLTEAPLNPK**	1953.06	652.03	-1.9	3	58.94	

Table 3. Identification of actin in the axial filament of *Monorhaphis sp.* glass sponge. The proteins extracted from the axial filament of *M.* sp were in-solution trypsin digested and analyzed by label-free nanoLC-MS/MS approach. Fifteen actin (A0A1Y9T597) peptides were identified with 40.2% sequence coverage.

Actin peptide sequence	Mass	m/z	Δ [ppm]	z	RT	Modification
**TTGIVFDSGDGVSHTVPIYEGYALPHAILR**	3184.62	797.16	-1.26	4	143.98	
**QEYDESGPSIVHR**	1515.7	506.24	2.43	3	52.1	
**VAPEEHPVLLTEAPLNPK**	1953.06	652.02	-2.13	3	89.33	
**AVFPSIVGRPR**	1197.7	400.23	-2.37	3	84.46	
**AGFAGDDAPR**	975.44	488.72	-2.93	2	44.63	
**GYSFTTTAER**	1131.52	566.76	-3.77	2	62.73	
**EITALAPPTMK**	1170.63	586.32	-0.16	2	85.87	
**DSYVGDEAQSKR**	1353.62	452.21	0.89	3	36.55	
**IIAPPER**	794.47	398.23	0.01	2	43.84	
**IIAPPERK**	922.56	308.52	2.04	3	34.82	
**GILTLK**	643.43	322.72	3.43	2	72.72	
**LDLAGR**	643.37	322.68	-1.01	2	48.44	
**IWHHTFYNELR**	1514.74	505.92	-2.71	3	74.97	
**DSYVGDEAQSK**	1197.51	599.76	-0.4	2	38.84	
**ILTER**	630.37	316.19	0.62	2	34.00	

Table 4. Identification of actin in the axial filament of *Caulophacus arcticus* glass sponge. The proteins extracted from the axial filament of *C. arcticus* were in-solution trypsin digested and analyzed by nanoLC-MS/MS approach. Twenty-nine actin (A0A1Y9T597) peptides were identified with 56.4% sequence coverage.

Actin peptide sequence	Mass	*m/z*	Δ [ppm]	z	RT	Modification
AGFAGDDAPR	975.44	488.72	-0.2	2	28.95	
DSYVGDEAQSK	1197.51	599.76	-1.2	2	25.65	
DSYVGDEAQSKR	1353.61	452.21	4	3	22.39	
IIAPPER	794.46	398.23	-2.4	2	31.69	
IIAPPERK	922.55	462.28	-0.6	2	23.24	
IWHHTFYNELR	1514.74	505.92	-1.3	3	42.64	
MTQIMFETFNTPAMYVAIQAVLSLYASGR	3252.60	814.15	-8.7	4	114.76	
QEYDESGPSIVHR	1515.69	506.23	-1.1	3	34.95	
QEYDESGPSIVHRK	1643.79	411.95	-2.7	4	26.79	
SYELPDGQVITIGNER	1789.88	895.94	-1.5	2	73.84	
AVFPSIVGR	944.54	473.27	-1.5	2	61.46	
AVFPSIVGRPR	1197.69	599.85	-1.2	2	50.46	
DLTDYLMK	997.47	499.74	-2.3	2	73.64	
GYSFTTTAER	1131.51	566.76	-0.2	2	40.16	
TTGIVFDSGDGVSHTVPIYEGYALPHAILR	3184.61	797.15	-3.2	4	83.84	
VAPEEHPVLLTEAPLNPK	1953.05	977.53	-2	2	58.69	
CDVDIRK	904.44	453.22	-1.3	2	20.92	Carbamidomethylation (2)
DLYANTVLSGGSTMFPGIADR	2200.04	1101.02	-2	2	83.65	Oxidation (14)
EKMTQIMFETFNTPAMYVAIQAVLSLYASGR	3557.72	1186.91	-3.8	3	109.41	Oxidation (3,7,16)
HQGVMVGMGQK	1186.55	396.52	0.8	3	22.53	Oxidation (20)
KYSVWIGGSILASLSTFQQMWISK	2745.41	916.14	1.4	3	109.78	Oxidation (20)
LDLAGRDLTDYLMK	1638.82	547.28	-1.7	3	70.47	Oxidation (13)
LDLAGRDLTDYLMKILTER	2251.18	751.40	0.8	3	97.33	Oxidation (13)
AGFAGDDAPR	975.44	488.72	-0.2	2	28.95	
DSYVGDEAQSK	1197.51	599.76	-1.2	2	25.65	
DSYVGDEAQSKR	1353.61	452.21	4	3	22.39	
IIAPPER	794.46	398.23	-2.4	2	31.69	
IIAPPERK	922.55	462.28	-0.6	2	23.24	
IWHHTFYNELR	1514.74	505.92	-1.3	3	42.64	

Table 5. Identification of actin and silicatein in the axial filament of *Spongilla lacustris* freshwater demosponge. The proteins extracted from the axial filament of *S. lacustris* were separated using the SDS-PAGE (**Figure S11**, Supporting Information) and analyzed by nanoLC-MS/MS approach. Three actin (A0A1Y9T597) and four silicatein (B5U9F0) peptides were identified with 12% and 19% sequence coverage, respectively.

Actin peptide sequence	Mass	*m/z*	Δ [ppm]	z	RT	Modification
VAPEEHPVLLTEAPLNPK	1953.07	652.02	3.2	3	53.46	
GYSFTTTAER	1131.52	566.76	0	2	37.51	
EITALAPPTMK	1170.63	586.32	2.7	2	47.82	
**Silicatein peptide sequence**	**Mass**	**m/z**	**Δ [ppm]**	**z**	**RT**	**Modification**
YVIDNGGIDTESSYSFK	1893.87	947.94	0.6	2	68.03	
NWGDSGYILMVR	1425.68	713.84	-1.3	2	63.93	Oxidation (10)
YQGQCGASYAFAARGALEGASALANDK	2691.22	898.08	-3	3	71.77	Carbamidomethylation (5)
FYQSGVFDSSSCSSTK	1785.75	893.88	2.5	2	54.78	Carbamidomethylation (12)

Table 6. List of actin binding and actin associated proteins identified in axial filaments of *Caulophacus arcticus* glass sponge. Actin, which has been identified as the main component of axial filaments in *C. arcticus* is presented in Supporting Information **Table 4**.

Protein ID	Protein name	Area	Part of total Area (%)
A0A1X7VLR9	Histone H4 OS=Amphimedon queenslandica	1.49E+09	7.07
A0A1X7V2V5	14_3_3 domain-containing protein OS=Amphimedon queenslandica	4.75E+08	2.26
Q7YZL7	Tubulin alpha chain (Fragment) OS=Aphrocallistes vastus	4.29E+08	2.04
O44348	Heat-shock protein Hsp70 (Fragment) OS=Rhabdocalyptus dawsoni	2.81E+08	1.33
Q7YZI6	90-kDa heat-shock protein (Fragment) OS=Aphrocallistes vastus	2.43E+08	1.15
A0A1X7V8F7	6-phosphogluconate dehydrogenase decarboxylating OS=Amphimedon queenslandica	2.16E+08	1.02
Q703I5	Gelsolin OS=Suberites ficus	2.02E+08	0.96
M1XMT6	Ras-related GTPase OS=Sycon ciliatum	1.79E+08	0.85
I2CCL8	Elongation factor 1 alpha (Fragment) OS=Bathydorus sp. SB-2012	1.75E+08	0.83

* **Histone H4** bundles F-actin, increases the viscosity of the F-actin containing solution and polymerizes G-actin.

* **14-3-3 domain** containing protein regulates actin filament formation.

*** Tubulin alpha chain**. The appearance of axial filament in demosponges, surrounding with microtubules in the sclerocyte cytoplasm at a very early stage of spiculogenesis has been observed using TEM.

* **Hsp70h** forms motile granules that are associated with actin microfilaments.

* **The 90 kDa** heat shock protein is a major phosphoprotein which associates various cellular polypeptides including actin.

***Gelsolin** belongs to a family of actin-severing and actin-capping proteins. It binds actin dimers and does nucleate polymerization. The orientation of monomers in the gelsolin nucleator is across the actin filament. Gelsolin also directly regulates actin dynamics in dendritic spines.

***RAS-related GTPase** promotes the formation of actin stress fibres.

* **Elongation factor-1 alpha** is an actin binding protein.

Table 7. **Comparison of axial filaments electron diffraction patterns.** The diffraction pattern of axial filaments identified in this study using FFT was compared with those described in literature (see also **Figure 3**)

Registered by us	Assignments for actin in the literature	Ref.
**1.80 ± 0.054 nm**	**1.85 nm**	82
**1.90 ± 0.05 nm**	**1.90 nm (**F-actin**)**	82, 86
**2.4 ± 0.07 nm**	**2.4 nm**	79,82,84
**3.4 ± 0.10 nm**	**3.4 nm**	82,87
**3.7 ± 0.11 nm**	**3.62 nm**	88
**4.6 ± 0.14 nm**	**4.9 nm**	72, 87
**5.3 ± 0.16 nm**	**5.4 nm (**F-actin)	89
**5.9 ± 0.18 nm**	**5.9 nm (**F-actin)	86,90-95
**11.1 ± 0.33 nm**	**11.1nm**	81
**12.9 ± 0.33 nm**	**12.5 ± 2.1 nm**<br>WT actin-actin spacing was 12.5 ± 2.1 nm 194 (mean ± SD; n=851), while Pls1-/- spacing was 12.7 ± 1.3 nm (mean ± SD; n=925).	95
**13.2 ± 0.40 nm**	**13.6 nm**<br>The distance D between the centres of neighbouring actin filaments	79
**14.0 ± 0.42 nm**<br>**14.3 ± 0.43 nm**	**14.5 nm**<br>adducin-f-actin complex	80
**38.5 ± 1.16 nm**	**37.8 nm**<br>A 28/13 symmetry, or ahelical repeat of 28 subunits in 13 turns of the 59A° pitch, would be 2.1538 units per turn, and the ‘‘crossovers’’ of the long-pitch helical strands would noccur every 378A°<br><br>**38 nm**<br><br>**38.5 nm**<br>Please note that Poole et al. 2006 registered peaks at 77 nm, 77nm/2 gives 38.5nm!<br><br>**39 nm**	75<br><br>76<br><br>78<br><br>77

Table 8: Position and assignment of Raman bands of actin.

Band position	Assignment	Band position	Assignment
621	Phe	1125	ν(C-C), ν (C-N)
642	Tyr	1156	ν(C-C), ν (C-N)
723	ν(C-S)	1173	δ (CH), Tyr
758	Trp	1207	Tyr, Phe
828	Tyr	1234	Amide III (β-sheets)
853	Pro Tyr	1250	Amide III (α-helix)
880	Trp	1266	Amide III (α-helix)
922	ν(C-C) Pro	1320	δ (CH_2)
933	ν(C-C) Pro	1340	Trp, δ(C-C_α-H), ν(C_α-C)
958	ν(C-C)	1448	δ (CH_2), δ (CH_3)
1003	Phe	1555	ν(C=C) Trp
1031	Phe	1585	δ (C=C) Phe Pro
1076	ν(C-C), ν (C-N)	1615	δ (C=C) Tyr Trp Phe
1099	ν(C-C), ν (C-N)	1666	Amide I

Supplementary references:

[61] Y. R. Lou, L. Kanninen, B. Kaehr, J. L. Townson, J. Niklander, R. Harjumäki, C. J. Brinker, M. Yliperttula, *Sci. Rep.* **2015**, *5*, 13635.

[62] K. R. Ayscough, J. Stryker, N. Pokala, M. Sanders, P. Crews, D. G. Drubin *J. Cell Biol.* **1997**, *137*, 399.

[63] I. Spector, N. R. Shochet, Y. Kashman, A. Groweiss, *Science* **1983**, *219*,493.

[64] P. Ganot, E. Tambutté, N. Caminiti-Segonds, G. Toullec, D. Allemand, S. Tambutté, *eLife* **2020**, *9*, e50022.

[65] R. Garrone, C. Lethias, In: *New perspectives in sponge biology. 3rd International Conference on the Biology of Sponges, 1985.* (K. Rützler), Smithsonian Institution Press, Washington, D.C. **1990**, pp. 121–128.

[66] T. L. Simpson, R. Garrone, M. Mazzorana, *J. Ultrastruct. Res.* **1983**, *85*, 159.

[67] M. J. Uriz, X. Turon, M. Becerro, *Cell Tissue Res.* **2000**, *301*, 299.

[68] T. L. Simpson, *The cell biology of sponges*, Springer-Verlag, New York, **1984**.

[69] J. Pottu-Boumendil, These. Univ. Claude Bernard, **1975**, pp. 1-101.

[70] G. Imsiecke, R. Steffen, M. Custodio, R. Borojevic, W. E. G. Müller, *In Vitro Cell Dev. An.* **1995**, *31*, 528.

[71] C. Levi, *C. R. Acad. Sci. Paris* **1963**, *256*, 497.

[72] C. Levi, *Cah. BioI. Mar.* **1964**, *5*, 97.

[73] D. P. Meka, R. Scharrenberg, B. Zhao, O. Kobler, T. König, I. Schaefer, B. Schwanke, S. Klykov, M. Richter, D. Eggert, S. Windhorst, *EMBO Rep.* **2019**, *20*, e47743.

[74] L. T. Nguyen, "Experimental structural characterization and MD simulation of F-actin self-assembly.", **2011**.

[75] A. Orlova, E. H. Egelman, *J. Mol. Biol.* **1994**, *245*, 582.

[76] H. Iwamoto, K. Oiwa, T. Suzuki, T. Fujisawa, *J. Mol. Biol.* **2001**, *305*, 863.

[77] F. van den Ent, L. A. Amos, J. Löwe, *Nature* **2001**, *413*, 39.

[78] K. J. V. Poole, M. Lorenz, G. Evans, G. Rosenbaum, A. Pirani, R. Craig, L. S. Tobacman, W. Lehman, K. C. Holmes, *J. Struct. Biol.* **2006**, *155*, 273.

[79] V. V. Lednev, D. Popp, *J. Struct. Biol.* **1990**, *103*, 225.

[80] K. A. Taylor, D. W. Taylor, *Biophys. J.* **1994**, *67*, 1976.

[81] A. A. Vazina, B. K. Lemazhikhin, G. M. Frank, *Dokl. Akad. Nauk SSSR* **1964**, *159*, 921.

[82] N. A. Zheleznaya, M. G. Mevkh, V. S. Gherasivov, *Naturwissenschaften* **1980**, *67*, 363.

[83] C. M. Hampton, D. W. Taylor, K. A. Taylor, *J. Mol. Biol.* **2007**, *368*, 92.

[84] J. C. Haselgrove, *J. Mol. Biol.* **1975**, *92*, 113.

[85] V. I. Risca, E. B. Wang, O. Chaudhuri, J. J. Chia, P. L. Geissler, D. A. Fletcher, *Proc. Natl. Acad. Sci. USA* **2012**, *109*, 2913.

[86] D. Popp, N. D. Loh, H. Zorgati, U. Ghoshdastider, L. T. Liow, M. I. Ivanova, M. Larsson, D. P. DePonte, R. Bean, K. R. Beyerlein, C. Gati, *Cytoskeleton* **2017**, *74*, 472.

[87] P. Matsudaira, J. Bordas, M. H. Koch, *Proc. Natl. Acad. Sci. USA* **1987**, *84*, 3151.

[88] D. S. Kudryashov, M. R. Sawaya, H. Adisetiyo, T. Norcross, G. Hegyi, E. Reisler, T. O. Yeates, *Proc. Natl. Acad. Sci. USA* **2005**, *102*, 13105.

[89] M. R. Sawaya, D. S. Kudryashov, I. Pashkov, H. Adisetiyo, E. Reisler, T. O. Yeates, *Acta Cryst.* **2008**, *D64*, 454.

[90] I. Matsubara, N. Yagi, H. Miura, M. Ozeki, T. Izumi, *Nature* **1984**, *312*, 471.

[91] E. H. Egelman, *J. Muscle Res. Cell Motil.* **1985**, *6*, 129.

[92] K. C. Holmes, D. Popp, W. Gebhard, W. Kabsch, *Nature* **1990**, *347*, 44.

[93] K. C. Holmes, W. Kabsch, *Curr. Opin. Struct. Biol.* **1991**, *1*, 270.

[94] H. E. Huxley, A. Stewart, H. Sosa, T. Irving, *Biophys. J.* **1994**, *67*, 2411.

[95] Z. Metlagel, *J. Struct. Biol.* **2019**, *206*, 149.
